# Poly(l‐Histidine)‐Mediated On‐Demand Therapeutic Delivery of Roughened Ceria Nanocages for Treatment of Chemical Eye Injury

**DOI:** 10.1002/advs.202302174

**Published:** 2023-07-10

**Authors:** Chia‐Jung Yang, Duc Dung Nguyen, Jui‐Yang Lai

**Affiliations:** ^1^ Department of Biomedical Engineering Chang Gung University Taoyuan 33302 Taiwan; ^2^ Department of Ophthalmology Chang Gung Memorial Hospital, Linkou Taoyuan 33305 Taiwan; ^3^ Department of Materials Engineering Ming Chi University of Technology New Taipei City 24301 Taiwan; ^4^ Research Center for Chinese Herbal Medicine College of Human Ecology Chang Gung University of Science and Technology Taoyuan 33303 Taiwan

**Keywords:** enhanced pharmacological bioavailability, eye injury therapy, multifunctional biomaterial system, smart drug delivery, surface engineering

## Abstract

Development of topical bioactive formulations capable of overcoming the low bioavailability of conventional eye drops is critically important for efficient management of ocular chemical burns. Herein, a nanomedicine strategy is presented to harness the surface roughness‐controlled ceria nanocages (SRCNs) and poly(l‐histidine) surface coatings for triggering multiple bioactive roles of intrinsically therapeutic nanocarriers and promoting transport across corneal epithelial barriers as well as achieving on‐demand release of dual drugs [acetylcholine chloride (ACh) and SB431542] at the lesion site. Specifically, the high surface roughness helps improve cellular uptake and therapeutic activity of SRCNs while exerting a negligible impact on good ocular biocompatibility of the nanomaterials. Moreover, the high poly(l‐histidine) coating amount can endow the SRCNs with an ≈24‐fold enhancement in corneal penetration and an effective smart release of ACh and SB431542 in response to endogenous pH changes caused by tissue injury/inflammation. In a rat model of alkali burn, topical single‐dose nanoformulation can efficaciously reduce corneal wound areas (19‐fold improvement as compared to a marketed eye drops), attenuate ≈93% abnormal blood vessels, and restore corneal transparency to almost normal at 4 days post‐administration, suggesting great promise for designing multifunctional metallic nanotherapeutics for ocular pharmacology and tissue regenerative medicine.

## Graphical abstract

A nanoceria with surface roughness control and poly(L‐histidine) coating is developed, which can be used for triggering multiple bioactive roles of intrinsically therapeutic nanocarriers and promoting transport across corneal epithelial barriers as well as achieving on‐demand drug release at the lesion site. Most importantly, single dose instillation of the nanoformulation effectively reduces corneal wound area and restores tissue transparency to normal.

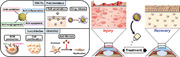

## 1 Introduction

Eye injuries are highly prevalent and impose enormous personal and socioeconomic impacts, which can occur in persons of all ages in any circumstances and become one of the major causes of blindness globally. It has been reported that, in the USA, there is an estimate of 24.5 million people suffering from the ocular insults; of these 1.5 million (≈6.1%) have experienced visual deficiency and ≈1.8 million (≈7.6%) are partially to totally blind.^[^ ^1^ ^]^ Among them, chemical corneal injuries are a prevalent trauma and responsible for 11.5–22.1% of the devastating ophthalmic emergencies.^[^ ^2^, ^3^ ^]^ Early signs and symptoms of this ocular disorder are pain, redness, irritation, tearing, foreign body sensation, and blurred vision; if not treated properly and timely, it can exacerbate and lead to severe complications such as corneal perforation, cataract, glaucoma, retinal damage, and visual loss.^[^ ^4^ ^]^ In particular, the exposure to strong acid or alkali often produced deep grade III injury.^[^ ^5^ ^]^ The first‐line treatment for chemical corneal injuries is topical instillation of anti‐inflammatory, anti‐oxidant, anti‐angiogenic, and anti‐fibrotic drugs or their combined dosage forms; ophthalmic solutions such as dexamethasone and rebamipide are the current medications for targeting the pathological factors.^[^ ^6^ ^]^ However, these conventional eye drops only can provide short‐term alleviation because of their low bioavailability caused by tear dilution, lacrimal drainage, and well‐known ocular barriers.^[^ ^7^, ^8^ ^]^ To circumvent these drawbacks, repetitive instillations of eye drop solutions with high dosages are required, which can cause systemic side‐effects not only in the eye but also other organs and tissues such as the heart, lung, or skin.^[^ ^9^ ^]^ Accordingly, development of topical formulations that can simultaneously address inflammation, oxidation, angiogenesis, and fibrosis while remarkably reducing the drug administration burden (i.e., enhancing pharmacological bioavailability) would be intensely beneficial for treating chemical corneal injuries.

Owing to their intriguing properties such as facile surface functionalization and controllable morphology/structure, nanoparticles (NPs) can be physically/chemically modified with various functional moieties, consequently opening new therapeutic strategies for clinical translation.^[^ ^10^, ^11^, ^12^ ^]^ Exploiting these modifiable features of NPs, ophthalmic nanoformulations can be elaborately engineered basing on particular biological scenarios to improve drug bioavailability at pathological sites.^[^ ^13^ ^]^ Of special interest is the manipulation of NP surface roughness for enhancing cellular uptake and thus improving drug delivery efficiency.^[^ ^14^, ^15^, ^16^, ^17^, ^18^ ^]^ It has been demonstrated that rambutan‐like silica NPs with a spiky rough surface possess superior adhesion in facilitating cellular internalization by examining the impact of their morphology on plasmid DNA transport.^[^ ^19^ ^]^ In addition, these rambutan‐like NPs reveal the highest plasmid DNA binding capability and transfection efficacy compared to those with other surface morphologies such as raspberry and flower‐like nanotopographies. Therefore, from the perspective of practical needs and considering that chemical corneal injuries can cause damage and deeper penetration into the stromal tissue, it is highly imperative to design nanocarriers with a properly rough surface for enhanced ocular bioavailability of topical drugs.

Ceria NPs have gained increasing attention due to their various bioactive functions.^[^ ^20^ ^]^ In ophthalmology, ceria NP has potential applications in treating diseases related to oxidative stress, inflammation, and neovascularization. Fiorani et al. have reported the successful use of ceria NPs to protect retinal cells from oxidative stress‐induced damage.^[^ ^21^ ^]^ As an effective anti‐inflammatory agent, ceria NP has been shown to ameliorate ocular surface inflammation by modulating inflammatory cytokine expression.^[^ ^22^ ^]^ Moreover, sustained inhibition of neovascularization via downregulating VEGF expression can be achieved by intravitreal injection of Ce NPs.^[^ ^23^ ^]^ Over the past few years, our group has reported the development of nanoceria for delivery of antiglaucoma medications.^[^ ^24^, ^25^, ^26^ ^]^ All the findings suggest that ceria NPs may hold promise to offer novel therapeutic strategies for various ocular disorders. Motivated by these encouraging outcomes, we further design new nanoceria‐based biomaterials for treating chemical eye injury. To effectively treat chemical corneal injuries via topical administration, the nanoformulations should be capable of penetrating through the corneal epithelium (the strongest ocular surface barrier), providing high drug bioavailability within the corneal tissue, and maintaining pharmacological effects in a sustained manner. A promising approach to offer potent tissue‐penetration capabilities is to combine nanocarriers with cell‐membrane penetrating moieties.^[^ ^27^, ^28^, ^29^ ^]^ Furthermore, nanocarriers can be chemically grafted with biofunctional substances that respond to innate biological stimuli such as pH, hypoxia, and enzymes for achieving superior drug bioavailability and sustained pharmacological activity at the lesion regions.^[^ ^30^, ^31^ ^]^ It is worth noting that the physiological environment of injured/inflamed tissues turned out to be acidic, possibly due to the damaged vasculature and the release of enzymes during phagocytosis.^[^ ^32^, ^33^ ^]^ In this context, poly(l‐histidine), which is not only able to penetrate through the cell membrane but also responsive to the endogenous pH signal, is sought as a resourceful surface moiety for pH‐responsive anticancer drug delivery^[^ ^34^ ^]^ and enhancing pharmacological performance of ophthalmic nanoformulations. This polypeptide can form positive charges in slightly acidic pH ranges and thus induce a solubility transition between healthy and injured microenvironments; it also can bind to the cellular membrane surface in a charge‐independent manner that enables cell/tissue permeability.^[^ ^32^, ^35^, ^36^ ^]^ Accordingly, a rational combination of surface‐roughed nanocarriers and poly(l‐histidine) can be crucially significant for the development of effective topical nanoformulations.

In this study, we develop a topical nanoformulation for the treatment of chemical corneal injuries via surface engineering of ceria nanocages. These NPs are rationally selected because of their modifiable surface roughness/functionality (tailorable biomedical properties), potential bioactive characteristics (anti‐oxidant, anti‐inflammatory, anti‐angiogenic, and anti‐apoptotic activities), and internal voids (“gigantic” drug containers). The NP surface roughness is regulated by harnessing the effects of etchant concentration during the synthesis of ceria nanocages. To boost the corneal tissue penetration and stimuli‐responsive release capabilities of the surface roughness‐controlled ceria nanocages (SRCNs), the NP surface is chemically integrated with poly(l‐histidine) at different amounts by an 1‐ethyl‐3‐(3‐dimethyl aminopropyl) carbodiimide hydrochloride (EDC) chemistry procedure. Acetylcholine chloride (ACh) and SB431542 are employed as model drugs for the SRCNs, as these medications can promote wound repair and prevent scar formation in the injured tissues. It is therefore hypothesized that an optimal surface roughness with an appropriate poly(l‐histidine) coating can endow the ACh/SB431542‐coloaded SRCNs with strong corneal epithelial permeability and sustained release performance while triggering multiple bioactive roles of ceria nanocages in stromal tissue repair, all of which can lead to an effective topical treatment of the ocular injuries.

## 2 Results and Discussion

### 2.1 Material Characterization and Biocompatibility

**Figure 1:**
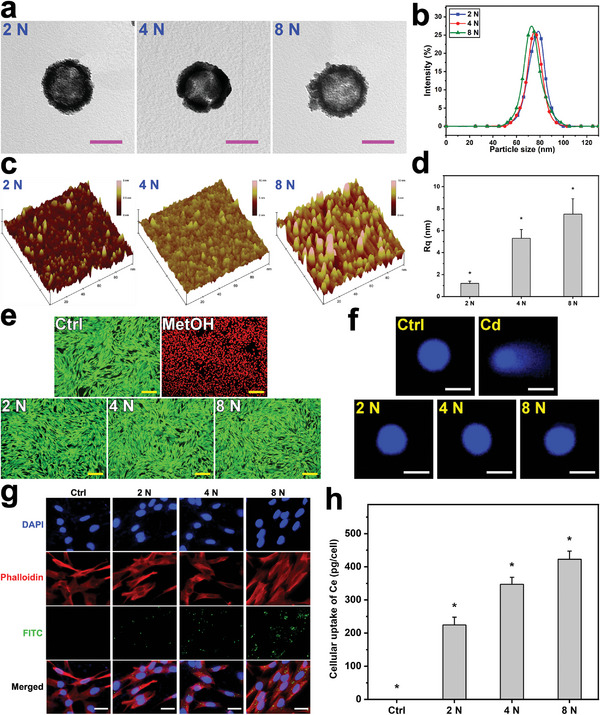
Material characterization and cytocompatibility studies. a) Typical TEM images, b) DLS spectra, c) surface textures, and d) mean square roughness of the SRCNs fabricated using different etchant concentrations. Values are mean ± SD (*n* = 5); *P < 0.05 versus all groups. Fluorescence photomicrographs of e) live/dead and f) comet assays of RCK cultures after a 2‐day exposure to various types of SRCNs. g) CLSM images of RCKs after incubation with various types of FITC‐labeled SRCNs for 4 h. h) Cellular uptake efficiency of the SRCNs. Values are mean ± SD (*n* = 5); *P < 0.05 versus all groups. Scale bars in a,e,f,g) are 50 nm, 50 µm, 10 µm, and 20 µm, respectively.

Our approach to formulate the ophthalmic nanoformulations featured with high corneal tissue permeability and sustained pH‐responsive drug release relies on the surface manipulation of the ceria nanocages through the controlled template‐etching reaction, PEGylation process, and amide bond formation (Figure S1, Supporting Information). To this end, SRCNs with an optimal surface roughness was first investigated. It is noteworthy that the etchant concentration is a crucial parameter to obtain SRCNs (Figure S2, Supporting Information). Solid silica@ceria NPs with relatively smooth surfaces could be observed in the untreated specimen (i.e., 0 N) while fragments (no nanocages) were found for the NPs treated at the highest concentration (12 N). The collapsed nanostructures can be attributed to the excess concentration that promotes rapid diffusion of etchant molecules to quickly dissociate the silica cores and thereby inducing an abrupt increase of water (a reaction product). In other words, the buildup of high pressure water within the NPs can trigger the collapse of ceria shells. On this basis, it is proposed that the employment of the etchant concentration in the range of 2–8 N can lead to the formation of ceria nanocages with tailorable‐rough surfaces (i.e., SRCNs). **Figure** **1**a shows typical transmission electron microscopy (TEM) images of SRCNs obtained after the treatment using different etchant concentrations (2, 4, and 8 N). All the SRCNs exhibited comparable lateral particle sizes (≈75 nm), but diverse surface roughness characteristics. In addition, dynamic light scattering (DLS) measurements showed no obvious difference in particle‐size distributions and diameters of the SRCNs (Figure 1b; Figure S3a, Supporting Information), further supporting the TEM data (Figure S2, Supporting Information). The degree of surface roughness of SRCNs was probed by atomic force microscope (AFM) and the root‐mean‐square roughness levels were analyzed accordingly (Figure 1c,d). The roughness degree is strongly dependent on the etchant concentrations, signifying that SRCNs with the highest surface roughness can be attained at the concentration of 8 N. The increase in surface roughness with increasing the etchant concentration can be ascribed to growing levels (higher concentrations lead to greater strengths) of the localized water pressure that outwardly pushes the ceria clusters/shells to become more protruded/dislocated (but not strong enough to initiate the collapse).

To examine whether SRCNs can be utilized as drug nanocarriers, their ocular biocompatibility was assessed in vitro by live/dead assays using rabbit corneal keratocytes (RCKs, the major cell type of the rabbit cornea) as model cells. All the SRCNs (2, 4, and 8 N) reveal good ocular biocompatibility as signified by the healthy and characteristic morphology and high mean percentage of RCKs in the cultures exposing to the NPs (Figure 1e; Figure S3b, Supporting Information). Possible genotoxicity of SRCNs was also evaluated by an alkaline comet assay. Fluorescence microscopic observation revealed that intact nucleus with smooth margin and no obvious comet formation (i.e., minimal DNA damage) could be found in the RCKs incubated with the SRCNs, demonstrating the negligible genotoxicity of the NPs (Figure 1f; Figure S3c, Supporting Information). Considering that surface roughness is a critical factor for regulating cell uptake of nanocarriers and thus the bioactive effects exerted by the ceria nanocages, cell internalization of different types of fluorescein isothiocyanate (FITC)‐labeled SRCNs was examined against RCKs (Figure 1g). Confocal laser scanning microscopy (CLSM) images indicated a gradual increase in the FITC signals (green color) around/within cell nuclei (blue color), implying that SRCNs with rougher surfaces could penetrate into cells at larger extents. This qualitative observation was further supported by comparing mean fluorescence intensities (Figure S3d, Supporting Information), confirming that the SRCNs (8 N) could be uptaken ≈1.5 and 6.8 times greater than SRCNs (4 N) and SRCNs (2 N), respectively. Furthermore, the amounts of SRCNs internalized by RCKs were quantitatively analyzed using inductively coupled plasma mass spectrometry (ICP‐MS) (Figure 1h). The masses of cerium uptake per cell for the SRCNs were 224.6 ± 23.4 pg cell^−1^ (2 N), 347.2 ± 21.4 pg cell^−1^ (4 N), and 423.0 ± 24.3 pg cell^−1^ (8 N). Previous studies have demonstrated high cellular uptake in the cultures exposed to the nanoparticles with high surface roughness.^[^ ^18^, ^37^, ^38^ ^]^ In particular, as indicated by others,^[^ ^39^ ^]^ the particles with higher surface roughness can lead to larger contact areas with cells, consequently facilitating their penetration into cells. Our findings are compatible with these earlier reports and imply that the underlying mechanism of cell contact with the nanoparticles may be related to the surface area of SRCNs. To elucidate the role of surface roughness in specific surface area of SRCNs, Brunauer‐Emmett‐Teller (BET) analysis was performed (Figure S4, Supporting Information). The specific surface areas were 5.3 ± 2.1 m^2^ g^−1^, 15.2 ± 1.6 m^2^ g^−1^, and 20.6 ± 2.8 m^2^ g^−1^ for 2 N, 4 N, and 8 N, respectively. It has also been reported that the increase in specific surface area can be achieved by the enhancement of surface roughness of silica nanoparticles.^[^ ^40^ ^]^ Collectively, these data demonstrate that all types of SRCNs are highly biocompatible to the corneal cells; cellular uptake of SRCNs increases with increasing their surface roughness, suggesting that bioactive effects of these nanocarriers can be improved with a highly roughed surface.

### 2.2 Bioactive Properties

**Figure 2:**
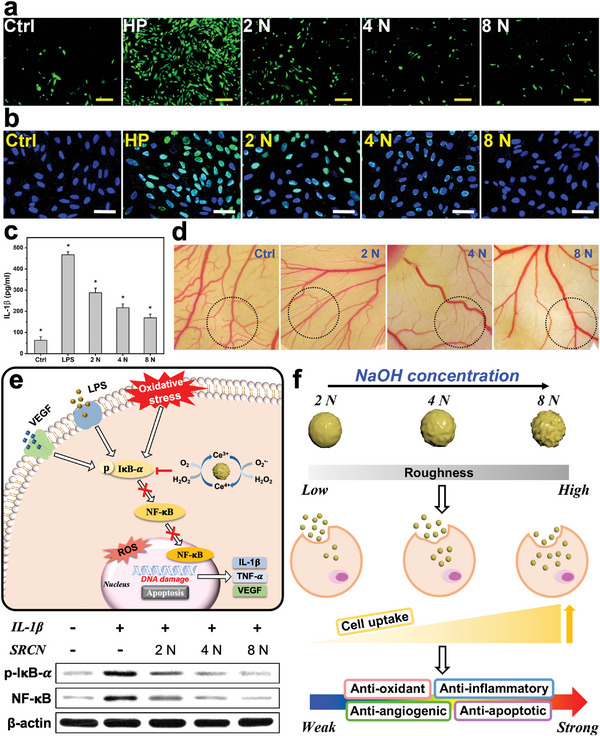
Bioactivity assays and mechanism of action studies. Effects of various types of SRCNs (2, 4, and 8 N) on suppression of H_2_O_2_ induced a) ROS production and b) cell apoptosis. RCKs were incubated with test SRCNs for 24 h and further exposure to H_2_O_2_ for 24 h; the cells incubated with no materials served as Ctrl. Levels of c) IL‐1β released from RCK cultures after incubation with various SRCN samples for 24 h. Values are mean ± SD (*n* = 5); *P < 0.05 versus all groups. d) Typical light micrographs of CAM vasculature after exposure to cellulose filter paper discs with tested SRCNs for 24 h. Dotted circles represent the areas of the CAM under the cellulose filter paper discs; Ctrl: cellulose filter paper discs without the SRCN pretreatment. e) Schematic illustration of the biological effects and mechanism of action via suppressing NF‐κB pathway of the SRCNs. Western blot analysis of p‐IκB‐α and NF‐κB expression in RCK cultures after a 24 h‐exposure to various SRCN samples. f) Demonstration of different types of SRCNs and their cellular uptake efficiencies and biological effects. Scale bars in a,b) are 50 and 100 µm, respectively.

Anti‐oxidant and anti‐apoptotic activities of SRCNs were evaluated in a cellular model of hydrogen peroxide‐induced oxidative stress (**Figure** **2**a,b; Figure S5a–c, Supporting Information). The results revealed that reactive oxygen species (ROS) productions in RCK cultures were inhibited by SRCNs in a surface roughness‐dependent manner (Figure 2a; Figure S5c, Supporting Information). Similarly, as the surface roughness increased, the calcium signal (blue fluorescence intensity) decreased (Figure S5a,b, Supporting Information); that is, rougher SRCNs could better reduce calcium overload in the RCKs. Furthermore, a terminal deoxynucleotidyl transferase (TdT)‐mediated dUTP nick end labeling (TUNEL) assay was employed to identify apoptotic cells caused by oxidative stress/calcium overload in RCK cultures incubated with SRCNs (Figure 2b; Figure S5d, Supporting Information). As expected, the pretreatment with SRCNs could reduce the cell apoptosis (green fluorescence signals) and superior anti‐apoptotic performances of SRCNs could be obtained in the groups with higher surface roughness. Our findings confirm the key role of highly roughed surfaces in better facilitating the internalization of the nanocarriers into cells and thereby triggering the anti‐oxidant and anti‐apoptotic activities therein.^[^ ^19^, ^41^ ^]^

To examine anti‐inflammatory activities of the SRCNs, the productions of interleukin‐1β (IL‐1β) and tumor necrosis factor‐α (TNF‐α) in lipopolysaccharide (LPS)‐induced inflammatory RCKs were analyzed, as these cytokines play significant roles in mediating the inflammation related to chemical corneal injuries.^[^ ^42^, ^43^ ^]^ The cytokine levels in the inflammatory cells treated with different SRCNs were gradually reduced with increasing surface roughness (Figure 2c; Figure S5e, Supporting Information). In addition, it is noting that inflammation‐induced angiogenesis plays a critical role in many ocular diseases (also including chemical burn), therefore inhibition of abnormal angiogenesis is considered an important therapeutic target for the treatment of corneal injuries. In this regard, anti‐angiogenic properties of SRCNs were assessed via their capability of suppressing vascular endothelial growth factor (VEGF)‐A_165_‐induced proliferation and migration of human umbilical vein endothelial cells (HUVECs) (Figure S5f–h, Supporting Information). The data indicated that rougher SRCNs exhibited stronger inhibitory activities against the proliferation and migration of the endothelial cells, which are essential in angiogenesis. The matrigel tube formation assay was executed to further evaluate the anti‐angiogenic properties of SRCNs since the formation of complete tube like structures from migrating endothelial cells is known to be a key step in angiogenesis.^[^ ^44^ ^]^ The tube formation is highest in SRCNs (2 N) followed by SRCNs (4 N) and SRCNs (8 N), implying the superior inhibitory effects could be obtained with the rougher nanocarriers (Figure S5i,j, Supporting Information). To support these, the chicken chorioallantoic membrane (CAM) assay was also conducted to quantify the vascular density index (Figure 2d; Figure S5k, Supporting Information). The diminution of branched blood vessels by SRCNs was observed to elevate with increasing the surface roughness; the vascular indexes of SRCNs (2 N), SRCNs (4 N), and SRCNs (8 N) were ≈46, 23, and 7%, respectively, confirming the surface roughness‐dependent anti‐angiogenic properties of SRCNs. These results suggest that anti‐inflammatory and anti‐angiogenic activities of the SRCNs can be improved by enhancing the intracellular therapeutic activities through increasing the surface roughness and thus cell uptake of the nanocarriers.^[^ ^19^, ^45^ ^]^

It is known that nuclear factor‐κB (NF‐κB) plays a crucial role in cell survival, cell proliferation, inflammation, and angiogenesis. The activity of NF‐κB is elevated with intracellular oxidative stress, which induce the phosphorylation and successive degradation of NF‐κB inhibitor‐α (IκB‐α) and thus enabling nuclear translocation of the free NF‐κB.^[^ ^46^, ^47^ ^]^ Nuclear NF‐κB can bind to its target sequence and thereby promoting transcription of a host of target genes such as IL‐1β, TNF‐α, and VEGF.^[^ ^48^ ^]^ Based on these, a schematic model on the SRCN inhibition of cell apoptosis caused by oxidative stress, inflammation, and angiogenesis through regulating a NF‐κB signaling pathway is proposed (Figure 2e). To inspect the influence of surface roughness of SRCNs on the signaling pathway mediating cell apoptosis, Western blot analysis was performed to identify phosphorylated form of IκB‐α (p‐IκB‐α) and NF‐κB expression (Figure 2e; Figure S5l,m, Supporting Information). The results indicated that surface roughness of the SRCNs is a key parameter in inhibiting the NF‐κB signaling in RCKs. Collectively, our data demonstrate that the etchant concentration‐mediated surface roughness of SRCNs is critically significant for achieving the intrinsically therapeutic nanocarriers with high cell uptake efficiency, consequently activating their intracellular therapeutic activities including anti‐oxidant, anti‐inflammatory, anti‐angiogenic, and anti‐apoptotic properties toward efficient treatment of injured cells/tissues (Figure 2f). In other words, these roughness effects can be ascribed to the stronger physical interactions between the rougher SRCNs and the biological entities, consequently facilitating the particles to penetrate into the cells and thereby triggering their bioactive activities therein at greater levels. On this basis, the SRCNs (8 N) with highest in vitro biomedical properties are selected for subsequent investigations.

### 2.3 Nanocarrier Functionalization and Permeability

**Figure 3:**
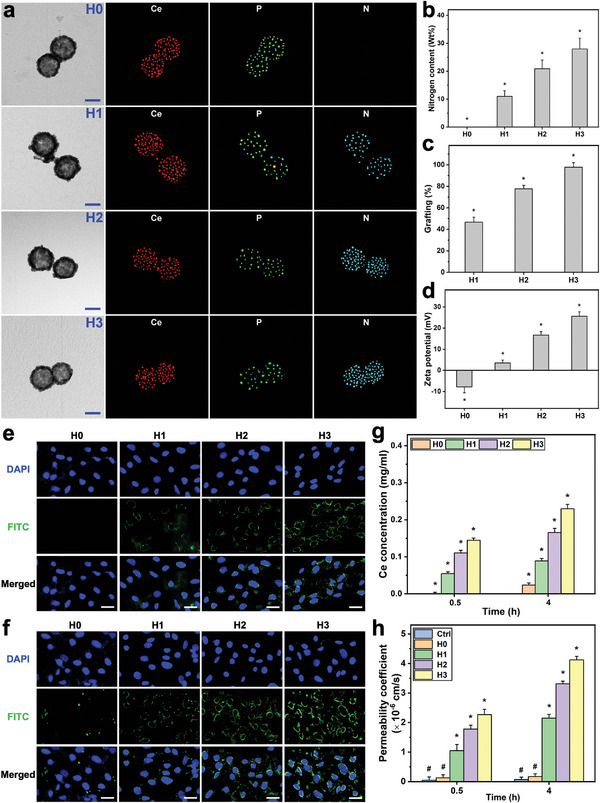
Nanocarrier functionalization and permeability studies. a) TEM images and corresponding EDS maps of cerium, phosphorus, and nitrogen of the PEGylated/functionalized SRCNs (H0, H1, H2, and H3 samples). Scale bars are 50 nm. b) The nitrogen content, c) amino group content, and d) zeta potential of the samples. Values are mean ± SD (*n* = 5); *P < 0.05 versus all groups. The CLSM images of RCKs treated with the samples for e) 0.5 h and f) 4 h. Scale bars are 20 µm. g) Cellular uptake efficiency of the various functionalized SRCNs. Values are mean ± SD (*n* = 5); *P < 0.05 versus all groups. h) Permeability coefficient of the samples. Values are mean ± SD (*n* = 5); *P < 0.05 versus all groups; ^#^ *P* < 0.05 versus H1, H2, and H3 groups.

The TEM images and their corresponding elemental energy dispersive X‐ray spectroscopy (EDS) maps implied that the polymer functionalization exerted no obvious impact on the ceria nanocage structures while presenting new chemical elements (P and N), which were further supported by quantitative chemical/physical analyses (**Figure** **3**a–d; and Section S2.2, Supporting Information). Furthermore, physical measurements and in vitro examinations proved the nanocarriers can be successfully functionalized with different poly(l‐histidine) amounts while maintaining their good ocular biocompatibility (Figures S6 and S7 and Section S2.2, Supporting Information).

To examine the effects of the polypeptide coatings on the cell penetration capabilities of the SRCNs, the transport of the functional nanocarriers through the rabbit corneal epithelial cells (RCECs) to RCK layers (i.e., mimicking the transport from the corneal epithelium to the stromal layer) was performed. The CLSM images (Figure 3e,f) revealed that the penetration capabilities of the functionalized SRCNs were increased with the polypeptide coating amounts, regardless of the incubation time. The relative penetration capabilities were further supported by quantitative analysis of the intracellular functional nanocarriers (Figure 3g,h; Figure S7e, Supporting Information), confirming the functionalized SRCNs (H3) possessed highest performance. In particular, the permeability coefficients of different functionalized SRCNs at 4 h post incubation were 2.15 ± 0.13 × 10^−6^ (H1), 3.31 ± 0.09 × 10^−6^ (H2), and 4.12 ± 0.12 × 10^−6^ (H3) cm s^−1^, all of which were remarkably greater than that without the polypeptide coating (H0, 0.17 ± 0.09 × 10^−6^ cm s^−1^). These findings signify that the highest poly(l‐histidine) amount (H3) can greatly improve the cell penetration capabilities of the SRCNs with an ≈24‐fold enhancement.

### 2.4 Drug Delivery and Pharmacological Activity

**Figure 4:**
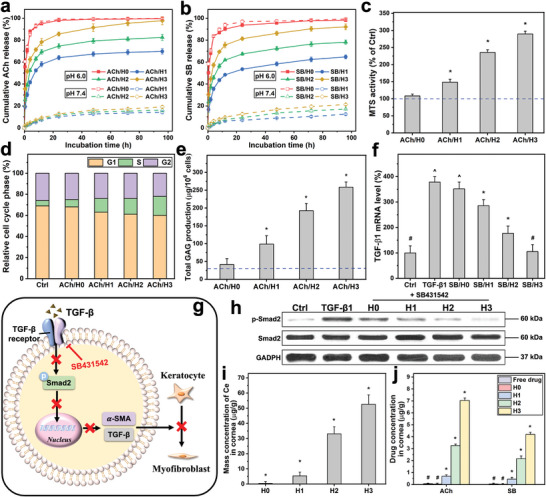
Drug delivery and pharmacological activity studies. Cumulative release percentage of a) ACh and b) SB from the samples at 34 °C in pH 6.0 and 7.4 solutions. Values are mean ± SD (*n* = 4). c) MTS activity, d) flow cytometry analysis, and e) total GAG content of RCKs exposed to the samples containing ACh for 2 days. The blue dash line represents the value for the cells cultured in the absence of test materials (Ctrl group). Results are expressed as percentage of Ctrl. Values in c) and e) are mean ± SD (*n* = 4); *P < 0.05 versus all groups. f) TGF‐β1 mRNA level of TGF‐β1‐induced RCK cultures treated with the samples containing SB. Values are mean ± SD (*n* = 5); *P < 0.05 versus all groups; ^#^ *P* < 0.05 versus TGF‐β1, SB/H0, SB/H1, and SB/H2 groups; ^^^ *P* < 0.05 versus Ctrl, SB/H1, SB/H2, and SB/H3 groups. g) Diagrammatic representation of the blockade of the TGF‐β induced Smad2 signaling pathway. h) Western blot analysis of p‐Smad2 and Smad2 expression in RCK cultures after a 2 day‐exposure to the samples containing SB. i) Ce and j) drug (ACh and SB) concentration in the cornea of injured eyes treated with the samples containing the drugs at 4 days post‐administration. Values are mean ± SD (*n* = 4); *P < 0.05 versus all groups; ^#^ *P* < 0.05 versus H1, H2, and H3 groups.

It is well‐known that the occurrence of oxidative stress in the cornea can be observed immediately after alkali injury.^[^ ^49^ ^]^ An antioxidant/prooxidant imbalance may further lead to excessive corneal inflammation, neovascularization, and fibrotic scarring formation.^[^ ^50^ ^]^ Hence, anti‐inflammation, re‐epithelialization, and anti‐fibrosis can each potentially contribute to the therapy outcome in the alkali‐burned corneas. Previous study has demonstrated that ACh facilitates corneal epithelial wound healing of rats.^[^ ^51^ ^]^ Furthermore, the pharmacological role of SB431542 as a specific inhibitor to the transforming growth factor‐β (TGF‐β) receptor has been elucidated.^[^ ^52^ ^]^ Based on our nanomedicine design, the administered functionalized SRCNs and released ACh/SB431542 can synergistically achieve the demanded pharmacological effects on anti‐inflammation and promotion of corneal re‐epithelialization as well as inhibition of scar formation, respectively, which offers a beneficial treatment modality for chemical eye injury. The evaluation of different functionalized SRCNs as biological stimuli‐responsive nanocarriers for the development of topical ophthalmic nanoformulations was conducted using ACh and SB431542 as model drugs, which are helpful to enrich the pharmacological efforts in the treatment of chemical corneal injuries. The method used for in vitro drug release studies was based on the pH‐responsive mechanism of poly(l‐histidine) (pKa ∼6.5) and acidic physiological environments (pH ∼6.0) of injured/inflamed interstitial tissues.^[^ ^53^, ^54^, ^55^ ^]^ During the loading process, the functionalized SRCNs were dispersed in the drug solution at acidic condition (i.e., pH 6.0). The poly(l‐histidine) coating on the nanocarrier surface was protonated and positively charged, generating an electrostatic repulsion to endow the SRCNs with opened structure that provided the access for diffusion of drug molecules into the hollow region of the nanoparticles.^[^ ^35^ ^]^ Upon pH adjustment to neutrality (i.e., 7.4), the poly(l‐histidine) chains were deprotonated and collapsed to form a protective covering layer on the surface of carrier system, and thus blocked the entrapped drug inside the SRCNs. The drug loading contents (Figure S8a, Supporting Information) and drug entrapment efficiencies (Figure S8b, Supporting Information) of the functionalized SRCNs exhibited increasing trends with increasing the poly(l‐histidine) coating amounts. This can be elucidated that higher surface coating amounts can wrap/seal the SRCNs more tightly in the neutral pH environment, consequently better confining the drug molecules within the nanocarriers. The cumulative drug release curves for ACh (**Figure** **4**a) and SB431542 (Figure 4b) displayed different profiles in response to the changes in pH values and polypeptide coating amounts. Rapid drug release characteristics in a pH‐independent fashion were observed for the nanocarriers without poly(l‐histidine) coating (H0). In contrast, for the functional nanocarriers (H1, H2, and H3), the release profiles were strongly dependent on the pH values and the biopolymer coatings. Less than 20% of the entrapped drugs were released at the neutral condition (pH 7.4) while 65–95% of those were detected in the acidic environment (pH 6.0) during a follow up period of 96 h; moreover, the higher polypeptide coating amount could mediate an increased release of both ACh and SB431542 from the SRCNs in controlled manners, attesting the substantial role of poly(l‐histidine) in response to pH changes. In addition, the drug amounts released from the functionalized SRCNs within 4 h exhibited a good accordance with the Higuchi model,^[^ ^56^ ^]^ suggesting the Fickian diffusion drug release mechanism of the two drugs in the acidic environment (Figure S8c,d, Supporting Information).

Prior to in vivo studies, it is necessary to assess in vitro pharmacological activities of ACh and SB431542 released from the functionalized SRCNs. Considering that ACh is implicated in promoting corneal stromal wound healing,^[^ ^57^ ^]^ the pharmacological effects of ACh released from the functional nanocarriers on the RCKs were studied. MTS assays (Figure 4c), flow cytometric analysis (Figure 4d; Figure S8e, Supporting Information), and extracellular matrix production assays (Figure 4e; Figure S8f, Supporting Information) suggest a promising strategy for improving corneal wound repair of ACh by rationally achieving an effective/increased release from the poly(l‐histidine)‐functionalized SRCNs (Section S2.3, Supporting Information).

In addition, during corneal wound healing, keratocytes exhibit a myofibroblastic phenotype (a source of fibrotic tissue in corneal scars) with respect to the TGF‐β, which is characterized by expression of α‐smooth muscle actin (α‐SMA).^[^ ^58^ ^]^ Accordingly, the pharmacological activity of SB431542 released from different functionalized SRCNs in preventing the TGF‐β1‐mediated transdifferentiation of keratocytes to myofibroblast was investigated. The results demonstrated that the released SB431542 could lower the elevated levels of TGF‐β1 mRNA (Figure 4f) and α‐SMA mRNA (Figure S8g, Supporting Information) in the TGF‐β1 stimulated RCKs; the reduction was proportionate to the SB431542 released from SRCNs, which was regulated by the polypeptide coating amounts (Figure 4b). Furthermore, it has been reported that TGF family members (including TGF‐β1) play their roles through the same signal transduction network such as Smad signaling pathway; binding to TGF receptors can cause the phosphorylation of signal transducer protein mothers against decapentaplegic homolog 2 (Smad2), and thus allowing this to translocate into the nucleus and regulating gene transcription.^[^ ^59^, ^60^ ^]^ Based on these earlier findings and the capability of the released SB431542 in reducing the high levels of TGF‐β1 mRNA and α‐SMA mRNA (excessive scarring), a schematic illustration for the blockade of the Smad2 signaling pathway is proposed (Figure 4g). To confirm this, Western blot analysis (Figure 4h) was carried out to detect p‐Smad2 and Smad2 expressions in RCK cultures exposed to various functionalized SRCNs containing SB431542. As anticipated, the relative protein levels of p‐Smad2/Smad2 (Figure S8h, Supporting Information) were found to decrease with respect to the increased release of SB431542 (via high polypeptide coating amounts). It is worth noting that the bioavailability of the therapeutic nanocarriers and drugs is proportional to the polypeptide coating amounts (Figure 4i,j; Figures S8i and S9, and Section S2.3, Supporting Information). According to literature reports, the minimum effective concentration of ACh to promote cell proliferation was 10 nm ^[^ ^57^ ^]^ while 140 nm ^[^ ^61^ ^]^ SB431542 was sufficient to inhibit TGF‐β‐induced fibrosis. At the end point (i.e., 4 day) of in vivo drug release studies, the concentrations of ACh and SB431542 were 48.1 and 10.9 µm, respectively (Figure 4j). Although the early release of surface‐bound drugs from nanocarriers is noted (Figure 4a,b), the concentrations of released drug at later time points remain above the therapeutic levels after topical application of 20 µl of the formulation to the ocular surface. Therefore, the SRCNs functionalized with highest poly(l‐histidine) coating amount were selected to examine their dosage form for pre‐clinical studies thereafter.

### 2.5 Pharmacotherapy of Corneal Burn

**Figure 5:**
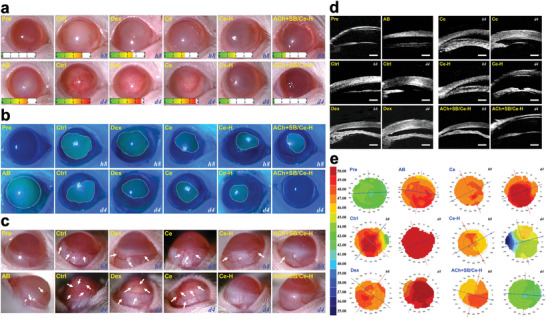
Clinical observations. a) Slit‐lamp biomicroscopic, b) corneal fluorescein staining, c) lateral view slit‐lamp biomicroscopic, d) ultrasound biomicroscopic, and e) corneal topography images of alkali burn (AB) eyes at 8 h and 4 d post‐instillation of ATS (Ctrl), dexamethasone eye drop (Dex), and ACh+SB/Ce‐H nanoformulation and its components including SRCNs (Ce) and poly(l‐histidine) functionalized SRCNs (Ce─H), respectively. Scale bars in d) are 500 µm. The score gauge in a) is the grade of corneal haze. A dotted white line in b) marks the margin of wound; white arrow in c) represents abnormal blood vessels.

To evaluate the treatment efficacy of the topical nanoformulation [poly(l‐histidine)‐functionalized SRCNs co‐loaded with ACh/SB431542], an experimental rat model of corneal alkali burn (AB) was employed. In vivo biocompatibility studies (Figure S10a–c and Section S2.4, Supporting Information) confirmed the safe use of the developed nanoformulations. **Figure** **5**a shows typical slit‐lamp biomicroscopic images of healthy (Pre), as‐induced AB (AB), artificial tear solution (ATS)‐treated AB (Ctrl), dexamethasone‐treated AB (Dex), and nanoformulation‐treated AB (Ce, Ce─H, and ACh+SB/Ce─H) eyes at 8 h and 4 d post‐instillation. Clinical manifestation of the AB eye was perceived in the AB group, as indicated by the smoky cornea and red conjunctiva. Trivial/low treatment efficacies were obtained for the injured eyes treated with ATS/Dex/Ce in a short time (8 h), probably due to the poor precorneal resistance of these dosage forms against tears and eye blinking (i.e., low bioavailability). With increasing the follow‐up to 4 days, all the pathological manifestations of AB became more evident. Treatment with the functional nanocarriers (Ce─H) could moderately alleviate the injured eyes, as verified by the reduction of redness and haziness. This observation elucidated that, owing to the assistance of the polypeptide, functional nanocarriers could penetrate into the diseased cornea and trigger the therapeutic activities of ceria to suppress inflammatory molecules and abnormal blood vessels. Notably, relatively high treatment efficiencies were achieved using the ACh+SB/Ce─H nanoformulation, as the treated AB eyes were recovered almost to healthy conditions such as vascularization‐free and transparent cornea and quiet anterior chamber. Quantitative analysis of corneal haze grade (Figure S10d, Supporting Information) further supports this qualitative observation. The effectiveness of ACh+SB/Ce─H can be ascribed to the synergistic combination of the potent bioactive properties of the ceria‐based nanocarriers and strong pharmacological activities of ACh and SB431542, all of which is regulated by the polypeptide coating.

In addition, ocular surface abnormalities and neovascularization of the AB eyes in response to different treatments were examined using fluorescein staining and slit‐lamp biomicroscopy, respectively (Figure 5b,c; Figure S10e,f, and Section S2.4, Supporting Information). Similar trends in the treatment efficacy were observed for the ACh+SB/Ce‐H nanoformulation. Specifically, the ACh+SB/Ce‐H revealed the highest performance with a 19‐fold improvement in the reduction of corneal wound areas compared to the marketed eye drops. It could also attenuate abnormal blood vessels (Figure 5c; Figure S10f, Supporting Information) at the strongest level (≈93% of the abnormal blood vessels were suppressed, compared to AB group), possibly due to the potent anti‐angiogenic properties of the ceria component.^[^ ^62^ ^]^ Ultrasound biomicroscopy and corneal topography measurements further confirmed the effectiveness of the ACh+SB/Ce‐H nanoformulation in restoring structural changes in the corneas caused by the chemical injury (Figure 5d,e; Figure S10g,h and Section S2.4, Supporting Information).

**Figure 6:**
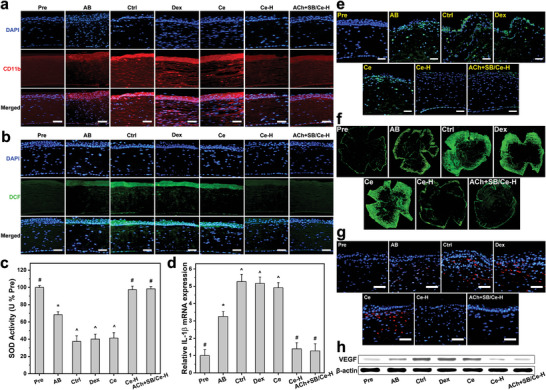
Therapeutic activity assays at 4 days post‐instillation. Immunofluorescence images of a) CD11b (red fluorescence)‐ and b) DCF (green fluorescence)‐stained corneas of AB rat eyes instilled with ATS (Ctrl), dexamethasone eye drop (Dex), Ce, Ce‐H, and ACh+SB/Ce‐H, respectively. c) SOD activity and d) IL‐1β levels in the corneal tissues of the treated AB eyes. e) Fluorescence images of different corneal sections stained with TUNEL (green fluorescence). f) Fluorescence images of flat‐mount whole corneal tissues; blood vessels are visualized by CD31 staining (green fluorescence). g) CD31 (red fluorescence) immunofluorescence staining images of different test corneal tissues. h) Western blot analysis of VEGF expression. Scale bars: 50 µm. Values are mean ± SD (*n* = 10); *P < 0.05 versus all groups; ^#^ *P* < 0.05 versus AB, Ctrl, Dex, and Ce groups; ^^^ *P* < 0.05 versus Pre, AB, Ce─H, and ACh+SB/Ce─H groups. The inclusion of the healthy (Pre) and as‐induced AB (AB) groups in all panels aims for comparative studies.

To further prove the effectiveness of the ACh+SB/Ce‐H nanoformulation in alleviating the AB eye through suppression of inflammation, oxidative stress, and neovascularization in the cornea, immunofluorescence staining and biochemical assays were performed. As shown in **Figure** **6**a, an obvious infiltration of CD11b (red fluorescence)‐positive inflammatory cells could be perceived in the injured cornea. The invasion of these inflammatory cells became more prevailing in the Ctrl, Dex, and Ce groups. In contrast, a substantial reduction of the CD11b fluorescence signal to comparable levels was observed in both Ce‐H and ACh+SB/Ce‐H groups, demonstrating the key role of the functional ceria component in suppression of corneal inflammation and further confirming the high efficacy of the nanoformulation in attenuation of the corneal edema. Interestingly, elevated ROS productions (superoxide and hydrogen peroxide) in the injured cornea were also demonstrated to be suppressed by both Ce‐H and ACh+SB/Ce‐H in a similar fashion as verified by dihydroethidium (DHE) (Figure S11a,b, Supporting Information) and dichlorofluorescein (DCF) (Figure 6b; Figures S11c, Supporting Information) staining data. Considering deficiency of antioxidants or excess of oxidants is implicated in chemical corneal injury,^[^ ^63^, ^64^, ^65^ ^]^ biochemical analysis was further employed to quantify levels of both antioxidants (superoxide dismutase: SOD and catalase: CAT) and oxidant maker (malondialdehyde: MDA) (Figure 6c; Figure S11d,e, Supporting Information) of the test eyes in response to the topical treatments. Administration of either Ce‐H or ACh+SB/Ce‐H could augment the antioxidant enzyme activities while lowering the oxidant marker levels of the injured eyes to close to those of the healthy animals. Compared to Dex, ATS, and Ce groups, the levels of SOD and CAT were approximately increased by 2.5 folds and those of MDA were decreased by ≈3.5 folds in Ce─H and ACh+SB/Ce‐H groups. In addition, real‐time reverse transcription polymerase chain reaction (RT‐PCR) was performed to investigate therapeutic effects of the topical nanoformulation on the mRNA expression of inflammatory cytokines including IL‐1β (Figure 6d) and TNF‐α (Figure S11f, Supporting Information). The results indicated a considerable increase in the inflammatory cytokine expressions in the AB corneas treated with ATS, Dex, and Ce formulations, suggesting their poor therapeutic performance. Topical instillation of Ce─H or ACh+SB/Ce─H significantly attenuated the AB‐induced elevation of the mRNA levels of IL‐1β and TNF‐α, attesting the critical role of poly(l‐histidine) coating in facilitating the transport of the therapeutic ceria component into the corneal stroma.

It has been reported that after stimulating the release of inflammatory cytokines, the ocular injuries can cause a rapid apoptosis of corneal cells.^[^ ^66^ ^]^ In this regard, a TUNEL assay was conducted to assess cellular apoptosis in the corneal tissues of the AB eyes with respect to different treatments (Figure 6e; Figure S11g, Supporting Information). The prominence of TUNEL (green fluorescence)‐labeled apoptotic cells was observed in AB group and was further enhanced in Ctrl, Dex, and Ce groups, as these instilled formulations possessed no therapeutic activities at 4 days post‐instillation. On the other hand, a remarkable reduction of the apoptotic cells was found in both Ce─H and ACh+SB/Ce─H groups, demonstrating the high effectiveness of the functional ceria component in preventing cell apoptosis via exerting their strong anti‐inflammatory and anti‐oxidant activities in the injured corneas.

Moreover, the therapeutic efficacy of the nanoformulation on suppression of corneal neovascularization was also assessed via immunohistochemical analysis of corneal whole flat mounts (Figure 6f; and S11h, Supporting Information) and corneal tissue sections (Figure 6g; Figure S11i, Supporting Information). The results showed that the healthy corneas were avascular while vascular areas were presented in AB group; the vascular areas were even more copious in AB eyes receiving the dosage forms without the polypeptide coating (e.g., Ce groups). Importantly, treatments using Ce‐H and ACh+SB/Ce‐H nanoformulations could mostly attenuate the vascular networks to the levels approaching those of healthy eyes. To support these immunohistochemical data, Western blot analysis (Figure 6h; Figure S11j, Supporting Information) was performed to determine the expression of VEGF, one of the most influential angiogenic factors in corneal neovascularization. Consistently, the ATS, Dex, and Ce formulations could not prevent the progression of VEGF‐related neovascularization in the injured eyes while Ce─H and ACh+SB/Ce─H were demonstrated to be capable of downregulating the elevated expression levels of the protein to normal.

**Figure 7:**
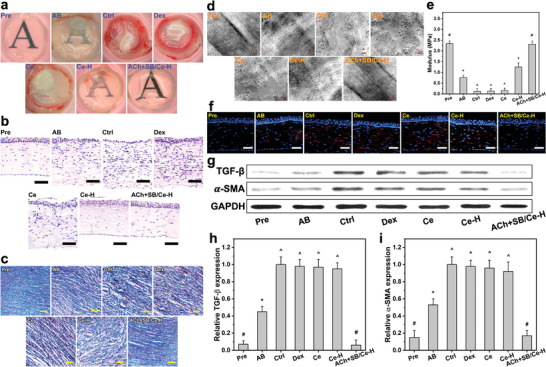
Tissue structure/function analyses at 4 days post‐instillation. a) Representative photographs of corneal tissues excised from AB rat eyes receiving ATS (Ctrl), dexamethasone eye drop (Dex), Ce, Ce─H, and ACh+SB/Ce─H, respectively. Corneal tissue sections stained with b) H&E and c) Masson's trichrome. Scale bars are 50 µm. d) Typical TEM images and e) Young's moduli of corneal stromal tissues from different test eyes. Scale bars in d) are 0.3 µm. Values are mean ± SD (*n* = 10); *P < 0.05 versus all groups; ^#^ *P* < 0.05 versus AB, Ctrl, Dex, Ce, and Ce─H groups; ^^^ *P* < 0.05 versus Pre, AB, Ce─H, and ACh+SB/Ce‐H groups. f) α‐SMA staining of the corneas from different test eyes. Scale bars are 50 µm. g) Western blot analysis of TGF‐β and α‐SMA expression. The relative expression level of h) TGF‐β and i) α‐SMA using ImageJ software. Values are mean ± SD (*n* = 10); *P < 0.05 versus all groups; ^#^ *P* < 0.05 versus AB, Ctrl, Dex, Ce, and Ce─H groups; ^^^ *P* < 0.05 versus Pre, AB, and ACh+SB/Ce─H groups. The inclusion of the healthy (Pre) and as‐induced AB (AB) groups in all panels aims for comparative studies.

Anatomically, corneal transparency relies on the well‐organized arrangement and orientation of collagen fibrils, thus any chemical injuries can induce tissue matrix remodeling in the cornea and cause a disrupted extracellular matrix.^[^ ^67^ ^]^ **Figure** **7**a displays typical photographs of the corneal tissues from healthy, as‐induced, and treated AB eyes. The ocular tissue of the Pre group was optically transparent while that of AB group exhibited haze features and dense blood vessels (red areas on the tissue margin). These vascular network characteristics developed more severely in Ctrl, Dex, and Ce groups, showing a good accordance with the staining results for corneal whole flat mounts (Figure 6f). Compared to the AB group, a reduction of blood vessels and an improvement in the optical transparence could be obtained in the Ce─H group. This can be ascribed to the effective penetration of the functional nanocarriers, consequently exerting an effect in terms of suppressing corneal neovascularization but missing to promote wound healing and prevent scar development. Notably, the instillation using the ACh+SB/Ce‐H nanoformulation could efficaciously restore corneal transparency of the AB eyes, as revealed by the normal avascular and see‐through characteristics of the treated corneal tissue. Furthermore, histological staining was performed to examine microscopic anatomy of the corneal tissues. The hematoxylin and eosin (H&E) stained tissues (Figure 7b) exhibited the multilayered tissue microstructures with highly disrupted integrity and swollen stromal layer in the AB and ATS/Dex/Ce‐treated corneas; these defects/disorders therefore acted as light scattering/absorbing sites to reduce the optical transparency of the corneal tissue (Figure 7a). The stromal edema and infiltrated inflammatory cells in the injured corneas could be mitigated by the administered Ce─H or ACh+SB/Ce─H, as signified by the well‐defined tissue integrity and structure. In addition, Masson's trichrome staining (Figure 7c) was utilized to evaluate the organization of collagen fibrils in the corneal tissues; noting that irregular collagen fibril arrangement is a cause of corneal edema and opaqueness.^[^ ^68^ ^]^ Similarly, the injured corneas possessed loose and disordered arrangement of the stromal collagen fibrils; this disrupted structure could be restored partially (Ce─H) to almost completely (ACh+SB/Ce‐H) by the poly(l‐histidine)‐based formulations. The remarkable restoration of the fibril alignments can be attributed to the capability of ACh in promoting the secretion of the collagen production (Figure S8f, Supporting Information), accordingly modulating extracellular matrix remodeling to rebuild matrix architectures.^[^ ^69^ ^]^

To further reveal the collagen fibril evolution in the injured stromal tissues with respect to different topical formulations, TEM analysis was performed (Figure 7d). Except ACh+SB/Ce‐H group, the stromal collagen fibrils in all treated groups were slackened and smashed; moreover, the fibril interspaces were markedly expanded (Figure S12, Supporting Information), indicating the damage of collagen structural arrangement and hence inducing corneal haziness (Figure 7a). Importantly, the ultrastructure of collagen fibrils in the ACh+SB/Ce‐H group exhibited densely and regularly packed collagen fibrils analogous to those of the healthy stromal tissue, showing a strong correlation with the good corneal transparency that is determined by highly ordered fibrils.^[^ ^70^ ^]^ Additionally, it has been reported that mechanical properties of the corneal tissue are mainly regulated by the collagen fibril structure and organization.^[^ ^71^ ^]^ In this regard, Young's modulus of the corneal tissues was measured (Figure 7e). The moduli for the tissues in Pre and AB groups respectively were 2.34 ± 0.13 and 0.76 ± 0.11 MPa, suggesting the weaker mechanical strength after the chemical trauma. As anticipated from the microstructure analyses, the corneal tissue in the ACh+SB/Ce─H group with a high recovery degree of fibril structure/alignment exhibited a mechanical strength (2.31 ± 0.15 MPa) comparable to that of the healthy tissue.

Considering that corneal haziness and fibrosis is characterized by the transdifferentiation of keratocytes into myofibroblasts,^[^ ^72^ ^]^ α‐SMA (a marker of mature myofibroblasts) staining of the corneal tissues was performed. As shown in Figure 7f, the high prevalence of α‐SMA‐positive cells (red fluorescence) in the AB/Ctrl/Dex/Ce groups signifies the generation of dense myofibroblasts in the corneal tissues, consequently causing severe corneal haziness in these groups (Figure 7a) as the transdifferentiated cells can secrete large quantities of disorganized extracellular matrix.^[^ ^73^ ^]^ To support these results, analysis of protein expressions of α‐SMA and TGF‐β was performed (Figure 7g–i), as the former is regulated by the latter.^[^ ^58^ ^]^ In a good accordance with the immunofluorescence staining data, the α‐SMA and TGF‐β expressions were high in the injured corneas and could not be downregulated by all instilled formulations, except ACh+SB/Ce─H. The noticeable reduction of the protein expressions by the ACh+SB/Ce─H nanoformulation can be ascribed to the SB431542, which is able to attenuate expression levels of α‐SMA and TGF‐β.^[^ ^74^ ^]^ It is noting that the ACh+SB/Ce─H nanoformulation also outperforms those using the SRCNs loading with either ACh or SB431542, possibly due to the synergistic amplification effects of the two drugs (Figure S13, Supporting Information). In order to assert a therapeutic effect due to the bioactive properties of SRCNs, it is important to compare the penetrated amount of ceria nanocage in the corneal tissues. In vivo bioavailability of SRCN nanocarriers was quantified using ICP‐MS. As compared to the Ce group, both the Ce─H and ACh+SB/Ce─H groups showed a significantly higher amount of nanomaterials in the corneal tissues (Figure S14a, Supporting Information), attesting the critical role of poly(l‐histidine) coating in facilitating the transport of the therapeutic ceria component into the corneal stroma. To further examine the in vivo fate of SRCNs following their ocular administration, the corneal tissue and aqueous humor specimens were collected at different follow‐up time points and analyzed to evaluate possible accumulation of ceria materials in vivo (Figure S14b, Supporting Information). Our results showed that the remaining amounts of SRCNs in both cornea and aqueous humor were gradually decreased with time. It was noted that the detected cerium concentrations in tissue specimens were almost zero at 10 days postoperatively, indicating the potential biosafety of the eye drop formulation. Collectively, the highest treatment efficacy of the ACh+SB/Ce‐H ophthalmic nanoformulation (Figure S15, Supporting Information) can be ascribed to the rational design/selection of the strong therapeutic SRCNs featured with effective penetration capabilities through corneal cells/tissues and endogenous stimuli release of both ACh and SB431542 (regulated by poly(l‐histidine)), thereby suppressing the oxidative stress/inflammation/neovascularization/apoptosis (by SRCNs), enhancing keratocyte growth (by ACh), and inhibiting the transdifferentiation of keratocytes to myofibroblast (by SB431542) in a synergistic manner.

## 3 Conclusion

In summary, we have developed an effective topical nanoformulation for the treatment of chemical corneal injuries by controlled manipulation of the surface roughness and therapeutic functionality of ceria nanocages in concert with synergistically pharmacological effects of ACh and SB431542. The surface roughness of ceria nanocages is demonstrated to be an important factor in elevating their therapeutic activities at cellular levels while the therapeutic functionality, mediated by the poly(l‐histidine) surface coating, can endow the nanoformulation with high corneal penetration capability and effective release of the dual drugs in response to the endogenous pH changes caused by the chemical trauma. The ophthalmic nanoformulation possesses good biocompatibility with the eye and provides sustained release of ACh and SB431542 for 4 days. In an experimental rat model of alkali burn, single dose instillation of the developed nanoformulation on the cornea could effectively mitigate the injured eyes. These findings suggest a promising route to develop effective topical nanoformulations with high tissue/cell penetration and endogenous stimuli‐responsive drug release for efficient management of chemical burns.

## 4 Experimental Section

### Controlled Fabrication and Characterization of SRCNs

To fabricate SRCNs, sacrificial nanosilica templates were employed owing to their high biocompatibility and wide uses in biomedical applications;^[^ ^75^, ^76^, ^77^, ^78^ ^]^ in a typical experiment, 300 mg of the sacrificial templates prepared using a sol‐gel method (Section S1.2, Supporting Information), were dispersed in ethylene glycol (45 ml) using ultra‐sonication. Then, 1 m cerium nitrate (2.25 ml) was added to the nanosilica dispersion followed by stirring for 10 min. The mixture was fixed in an autoclave and heated to 130 °C for 6 h to obtain ceria‐coated nanosilica particles. After cooling, the ceria‐coated nanosilica particles were separated by centrifugation (22 000 × g, 10 min) followed by washing with excess ethanol. The fabrication of different types of SRCNs was carried out by dispersing the ceria‐coated nanosilica particles in sodium a hydroxide solution at different concentrations (0–12 N) for 2 days at ambient conditions; a corresponding fresh etchant solution was substituted after the first day. Finally, different SRCN samples were achieved by consecutive centrifugation (10 000 × g, 10 min), re‐dispersion in water and then ethanol, and drying in air. The SRCNs were morphologically and structurally characterized using a TEM (JSM‐1200EX II JEOL). Moreover, DLS measurements of the SRCNs was conducted using a Doppler microelectrophoresis (Zetasizer Nano ZS, Malvern Instruments, Worcestershire, UK) and their average diameters were calculated (*n* = 5). The surface texture of the SRCN samples were examined with an AFM (Bruker, Billerica, MA, USA).

### Cytocompatibility

All procedures for animal experiments herein were approved by the Institutional Animal Care and Use Committee of Chang Gung University (Approval Number: CGU109‐173) and were executed in agreement with the ARVO Statement for the Use of Animals in Ophthalmic and Vision Research. Corneal stromal tissues of twenty female adult New Zealand white rabbits (National Laboratory Animal Breeding and Research Center, Taipei, Taiwan, ROC) with weights of 2.5–3.0 kg were used to obtain rabbit corneal epithelial cells (RCECs) and keratocytes (RCKs) with proper morphology and phenotype^[^ ^79^ ^]^ for in vitro experiments. The cytocompatibility of SRCNs was evaluated using Live/Dead and comet assays (please see Section S1.3, Supporting Information for more details).

### Cellular Uptake Efficiency Studies

The FITC‐labeled SRCNs were used to investigate cellular uptake efficiencies using a CLSM (Leica, Heidelberg, Germany) and ICP‐MS (Agilent Technologies, Tokyo, Japan), respectively (please see Section S1.4, Supporting Information for more details).

### Bioactivity Assay Studies

Antioxidant activities of the SRCNs were assessed via their capabilities in reducing the elevated levels of intracellular ROS and intracellular calcium in RCK cultures. In addition, anti‐apoptotic activities of SRCNs against ROS‐induced cell apoptosis were studied using a TUNEL assay (Roche Diagnostics, Indianapolis, IN, USA). Enzyme‐linked immunosorbent assays (ELISA, R&D Systems, Minneapolis, MN, USA) were employed to examine anti‐inflammatory activities of the SRCNs through detection of IL‐1β and TNF‐α from inflamed RCKs. Anti‐angiogenic properties of the SRCNs were evaluated using VEGF‐induced HUVEC proliferation, cell migration, and CAM assays. Moreover, Western blot was employed to inspect bioactivity mechanism of action of SRCNs on the signaling pathway mediating cell apoptosis. Please see Section S1.5, Supporting Information for more details.

### Functionalization of SRCNs and Cytocompatibility Studies

Details on the functionalization and characterization of SRCNs with different poly(l‐histidine) amounts can be found in Section S1.6 (Supporting Information). Cytocompatibility studies were conducted using similar methods reported in Section 2.2.

### Penetrating Capability Studies

RCECs were employed to explore a possible permeability of the functionalized SRCNs to stromal cells from epithelial cells. Details please see Section S1.7 (Supporting Information).

### In Vitro and In Vivo Therapeutic Studies

Phase‐contrast microscopy, cell proliferation MTS assay, and ELISA assays, high performance liquid chromatography (HPLC) analysis, and slit‐lamp biomicroscopy were used to evaluate the in vitro and in vivo therapeutic properties of the hydrogel‐based systems. Further information can be found in Section S1.8 (Supporting Information).

### In Vitro Drug Release Studies

ACh and SB431542 were utilized as the two model drugs for the investigation of their release profiles from SRCNs. Drug loading content (%) and entrapment efficiency (%) were calculated as follows: loading content (%) = (*W*_t_/*W*_np_) × 100% and entrapment efficiency (%) = (*W*_t_/*W*_i_) × 100%, where *W*_t_ is the total weight of the drug confined in the nanocarriers, *W*_np_ the weight of the nanocarriers, and *W*_i_ the initial weight of the drug mixed with the nanocarriers (*n* = 4). Please see Section S1.8 (Supporting Information) for more details.

### Pharmacological Activity Studies

Pharmacological activities of ACh released from the functionalized SRCNs were studied by a cell proliferation MTS assay, flow cytometry, and extracellular matrix production assay. On the other hand, ELISA and Western blot were employed to examine anti‐fibrotic activities of the released SB431542. In vivo bioavailability of SRCN carriers and drugs in the corneal tissues was quantified using ICP‐MS and HPLC, respectively. Please see Section S1.9 (Supporting Information) for more details.

### In Vivo Biocompatibility Studies

A slit‐lamp biomicroscope (Topcon Optical, Tokyo, Japan), H&E staining, and a light microscope (Carl Zeiss) were used to assess in vivo biocompatibility of the SRCN‐based nanoformulations. Please see Section S1.10 (Supporting Information) for more details.

### In Vivo Therapeutic Studies

A round filter paper (≈4 mm in diameter) was soaked in 1 N NaOH solution and then applied on the central corneal surface for 30 s. The ocular surface was then rinsed with 30 ml of a sterile normal saline. A total of 50 rats were used. For the four test groups (Dex, Ce, Ce─H, ACh+SB/Ce─H) of animals (10 rats/group), the AB rats received single topical instillation of 20 µl of dexamethasone (Dex, 0.1%) or each of SRCN‐based formulations including Ce (20 µg Ce), Ce─H (20 µg Ce and 20 µg H), and ACh+SB/Ce─H (20 µg Ce, 20 µg H, 6.12 µg ACh, and 4.66 µg SB). Ten AB rats instilled with an ATS (20 µl) served as Ctrl group. At preoperation (Pre), all the rats were healthy without clinically observable ocular surface disorders. Corneal haziness, corneal epithelial defects, corneal thickness, and corneal topography of the test eyes were analyzed to assess therapeutic effects of the test nanoformulations (please see Section S1.11, Supporting Information for more details).

### Therapeutic Activity Studies

Immunofluorescence staining and biochemical assays were carried out to study in vivo therapeutic activities of the developed nanoformulations. Specifically, CD11b immunostaining and real‐time RT‐PCR were respectively conducted with the corneal tissues collected from the enucleated rat eyes for in vivo anti‐inflammatory activities. On the other hand, in vivo anti‐oxidant properties were assessed using DHE staining and biochemical assays including SOD, CAT, and MDA levels. Apoptotic corneal cells in the tissue sections were examined by TUNEL assay (Roche Diagnostics) for the assessment of in vivo anti‐apoptotic activities. Moreover, the suppression of corneal neovascularization by the nanoformulations was assessed via immunohistochemical analysis of corneal whole flat mounts and corneal tissue sections. Western blot analysis was carried out to further support the immunohistochemical data. Please see Section S1.12 (Supporting Information) for more details.

### Tissue Structure/Function Analysis

The corneal tissues excised from the test eyes were digitally imaged for gross appearance, stained with H&E and Masson's trichrome for estimation of total corneal thickness and collagen organization, and processed for ultrastructural observation by TEM as well as Young's modulus measurement. Additionally, α‐SMA (a marker of mature myofibroblasts) staining of the corneal tissues and Western blot were implemented for anti‐fibrotic function analysis. Please see Section S1.13 (Supporting Information) for more details.

### Statistical Analysis

Results were expressed as mean ± standard deviation. Comparative studies of means were performed using one‐way analysis of variance (ANOVA). Significance was accepted with *P* < 0.05.

Detailed experimental materials and methods can be found in the Supporting Information.

## Conflict of Interest

The authors declare no conflict of interest.

## Supporting information

Supporting Information

## Data Availability

The data that support the findings of this study are available from the corresponding author upon reasonable request.
