# Supplementary material for: Poly(l‐Histidine)‐Mediated On‐Demand Therapeutic Delivery of Roughened Ceria Nanocages for Treatment of Chemical Eye Injury
Source: Adv Sci (Weinh). 2023 Jul 10;10(26):2302174. doi: 10.1002/advs.202302174 (PMC10502830; doi:10.1002/advs.202302174)
Supplement: Supplementary file 1 — Supporting Information [file ADVS-10-2302174-s001.pdf]

ADVANCED SCIENCE
Open Access

Supporting Information

for *Adv. Sci.*, DOI 10.1002/advs.202302174

Poly(L-Histidine)-Mediated On-Demand Therapeutic Delivery of Roughened Ceria Nanocages for Treatment of Chemical Eye Injury

*Chia-Jung Yang, Duc Dung Nguyen and Jui-Yang Lai**

ADVANCED SCIENCE
Open Access

Supporting Information

for *Adv. Sci.*, DOI 10.1002/advs.202302174

Poly(L-Histidine)-Mediated On-Demand Therapeutic Delivery of Roughened Ceria Nanocages for Treatment of Chemical Eye Injury

*Chia-Jung Yang, Duc Dung Nguyen and Jui-Yang Lai**

Supporting Information

Title
Poly(L-Histidine)-Mediated On-Demand Therapeutic Delivery of Roughened Ceria Nanocages for Treatment of Chemical Eye Injury

*Chia-Jung Yang, Duc Dung Nguyen, and Jui-Yang Lai**

1. Supplemental Experimental Section

1.1. Materials

Cerium (III) nitrate hexahydrate (99% trace metal basis), tetraethyl orthosilcate (TEOS), ethanol (anhydrous > 99.5%), ethylene glycol (anhydrous 99.8%), ammonium hydroxide (28% NH_3 in H_2O), sodium hydroxide (97%), 1-ethyl-3-(3-dimethyl aminopropyl) carbodiimide hydrochloride (EDC), poly(L-histidine), ninhydrin reagent, lipopolysaccharide (LPS), ACh, and SB431542 were all purchased from Sigma-Aldrich (St. Louis, MO, USA). Phosphonate-PEG-COOH (MW of 1200 g/mol) was bought from Specific Polymers (Castries, France). Deionized (DI) water was prepared with a purification Milli-Q system (Millipore, Bedford, MA, USA). Balanced salt solution (BSS, pH 7.4) was supplied by Alcon Laboratories (Fort Worth, TX, USA). All the other chemicals were of reagent grade and used as purchased.

1.2. Synthesis of Nanosilica Templates

A sol-gel method was first adopted to synthesize nanosilica templates. Briefly, TEOS (8 ml) was mixed with ethanol (280 ml), and the resultant mixture was kept under a stirring condition at room temperature. Subsequently, DI water (56 ml) and ammonium hydroxide (8.4 ml) were added to the mixture; the resultant solution was retained in a sealed flask at room temperature under a continuous stirring for 24 h. After that, the solution was

consecutively centrifuged (10000 × g, 10 min) and re-dispersed in water and ethanol, and finally dried at 65 °C for 6 h in an oven to obtain nanosilica templates.

1.3. In Vitro Biocompatibility Studies

The corneal stromal tissues with epithelium, harvested by aseptically stripping their Descemet's membrane with the attached endothelium using a dissecting microscope (Leica, Wetzlar, Germany), were incubated in 5 mg/ml of dispase at 4 °C overnight. Subsequently, loose epithelial layers were discarded, and the cells were maintained with supplemental hormonal epithelial medium (SHEM), consisting of an equal volume of HEPES-buffered DMEM containing bicarbonate and Ham's F-12, 0.5% DMSO, 2 ng/ml mouse epidermal growth factor, 5 µg/ml insulin, 5 µg/ml transferrin, 5 ng/ml selenium, 0.5 µg/ml hydrocortisone, 30 ng/ml cholera toxin A subunit, 5% FBS, 50 µg/ml gentamicin, and 1.25 µg/ml amphoterincin B. To isolate the cells, the stromal discs were chopped into small pieces and digested using 4 mg/ml collagenase at 37 °C for one day. Single stromal cells were mechanically separated from clumps of the processed stroma pieces and maintained in a regular culture medium consisting of DMEM/ F12, 10% FBS, and 1% A/A solution. All the cell cultures were incubated in a humidified atmosphere containing 5% CO_2 at 37 °C. The medium was freshly replaced every other day. Confluent cell layers were subcultured by trypsin-EDTA at a 1:4 split ratio. The second-passage rabbit corneal epithelial cells (RCECs) and keratocytes (RCKs) were employed in all *in vitro* experiments.

RCKs (5×10^4 cells/well) were seeded into 24-well plates by 1 ml/well. Prior to testing, the SRCNs were sterilized in with 70% ethanol. 20 µl of various SRCNs (H0: 20 µg Ce; H1: 20 µg Ce + 5 µg H; H2: 20 µg Ce + 10 µg H; H3: 20 µg Ce + 20 µg H) were added to the regular growth medium and incubated for 2 days. RCK cultures without exposing to the SRCNs served as Ctrl groups. Cell viability was conducted using a Live/Dead Viability/Cytotoxicity Kit (Molecular Probes, Eugene, OR, USA) and a fluorescence microscope (Axiovert 200M; Carl Zeiss, Oberkochen, Germany). The live cells were

recognized through green fluorescence signals from the intracellular esterase activities due to cleavage of calcein acetoxymethyl. In contrast, dead cells identified by red fluorescence signals generated via the binding of EthD-1 to the nucleic acids in dead cells with damaged cell membranes. Furthermore, the genotoxicity of the SRCNs to RCKs was assessed by the comet assay. After lysing cells, the slides were positioned on a horizontal gel electrophoresis platform and covered with an alkaline solution containing 300 mM NaOH and 1 mM EDTA (pH 13). DNA was permitted to unwind for 1 h, and then an electric field (300 mA, 21 V) was applied at 4 °C for 40 min. Subsequently, the slides were stained with 4’,6-diamidino-2-phenylindole (DAPI; Vector Laboratories, Peterborough, England) for imagining under the fluorescence microscope. The results measured from 50 random comets per slide were averaged. The DNA damage for each cell was quantified as follow: comet tail length (μm) = maximum total length – head diameter. Data were averaged based on 4 independent runs.

1.4. Cell uptake studies

For fluorescent labeling, SRCNs were first surface-modified with phosphonate-PEG-COOH, followed by reaction with ethylenediamine to allow the formation of amine-terminated surface. Subsequently, the ethylenediamine functionalized SRCNs were dispersed in ethanol containing EDC, and 1.0 mg of fluorescein isothiocyanate (FITC) was added. The reaction mixture was continuously stirred for 24 h in dark conditions. The solid particles were separated by centrifugation and washed with excess ethanol. The final products were achieved after drying the clean solid particles at 45 °C in vacuum conditions.

The FITC-labeled SRCN samples (1 mg/ml) were added to the RCK culture plates (cell density of 1×10^5 cells/well) of each test group and incubated for 4 h at 37 °C. A confocal laser scanning microscope (CLSM, Leica, Heidelberg, Germany) was used for imaging after staining the nuclei with DAPI and the cytoskeleton with rhodamine-phalloidin. The blue fluorescence of DAPI and the red fluorescence of phalloidin were respectively observed using a 405 nm and 540 nm laser source while the green fluorescence of the FITC-labeled SRCNs

was observed by using a 488 nm laser light and its fluorescence intensity was quantified accordingly.

The cells were then washed with a phosphate-buffered saline (PBS) three times and collected with trypsin. After a centrifugation process, the cell pellets were washed twice and dried. A cell lysis buffer (Cell Signaling Technology) was added to allow dissociation of the cells by sonication. The supernatants (containing cell components) were discarded by centrifugation at 16000 × g for 5 min, and the precipitated solid was washed with PBS twice. The Ce concentrations in the final solutions were measured using inductively coupled plasma mass spectrometry (ICP-MS, Agilent Technologies, Tokyo, Japan) (n = 5). The specific surface area of SRCNs was determined by the nitrogen adsorption test using Brunauer-Emmett-Teller (BET) technique with Micromeritics ASAP 2010 equipment.[(1)] Data were averaged from five independent experiments.

1.5. Bioactivity Assay Studies

1.5.1. In Vitro Anti-Oxidant Activity Studies

To evaluate the anti-oxidant activities of different types of SRCNs, RCK cultures exposed to 0 (Ctrl group) or 200 (HP group) μM H_2O_2 for 24 h without 24 h of pretreatment with any substances were employed for comparison. Intracellular accumulation of ROS was estimated by oxidative conversion of cell-permeable 2’,7’-dichlorodihydrofluorescein diacetate (DCFH-DA) (Molecular Probes) to fluorescent 2’,7’-dichlorofluorescein (DCF). The RCK cultures were incubated with a 10 μM DCFH- DA solution at 37 °C for 1 h. The cultured cells were then washed three times with PBS. The DCF fluorescence imaging (Ex. 488 nm; Em. 525 nm) was acquired with the fluorescence microscope. Furthermore, the fluorescence reading was completed with a multimode microplate reader (BioTek Instruments, Winooski, VT, USA) to check the difference in the fluorescence intensity (n = 3). Intracellular overload of calcium was assessed exploiting Fura-2, AM (Molecular Probes), a Ca^{2+}-sensitive fluorescent indicator. In particular, the RCKs were incubated with a 5 μM

Fura-2, AM solution at 37 °C for 1 h. After that, the cells were washed three times with PBS. The fluorescence imaging (Ex. 340 nm; Em. 510 nm) was obtained using the fluorescence microscope. To quantify intracellular calcium content, the cells were gathered and redispersed in *N*-2-hydroxyethylpiper-azine-*N'*-2-ethanesulfonic acid (HEPES)-buffered saline solution (containing 132 mM NaCl, 2 mM $CaCl_2$, 3 mM KCl, 10 mM glucose, and 10 mM HEPES, pH 7.4). The fluorescence reading was obtained using the multimode microplate reader to probe the change in the fluorescence intensity (n = 3).

1.5.2. In Vitro Anti-Apoptotic Activity Studies

Anti-apoptotic activities of various types of SRCNs were assessed including the RCK cultures exposed to 0 (Ctrl group) or 400 (HP group) μM H_2O_2 for 24 h without 24 h of pretreatment with any materials were used for comparison. Apoptotic cells in the cell cultures were determined using a terminal deoxynucleotidyl transferase (TdT)-mediated dUTP nick end labeling (TUNEL) assay (Roche Diagnostics, Indianapolis, IN, USA). To this end, samples were fixed with 4% paraformaldehyde at room temperature for 1 h. The fixed specimens were washed with PBS and then permeabilized in 0.1% Triton X-100 in 0.1% sodium citrate for 2 min on ice, followed by incubating with a mixture of TdT and fluorescein isothiocyanate dUTP solutions in a humidified chamber at 37 °C for 1 h. The samples were stained with the DAPI and examined microscopically at a 400× magnification for the fluorescent visualization of cell nuclei. Ten different regions were arbitrarily chosen for quantitative analysis of TUNEL-positive cell nuclei. The apoptotic index was calculated as the average ratio of apoptotic cells to the total cells in these 10 different areas (n = 6).

1.5.3. In Vitro Anti-Inflammatory Activity Studies

Anti-inflammatory activities of the SRCN samples were evaluated by enzyme-linked immunosorbent assays (ELISA). RCKs with a density of 5×10^4 cells/well were incubated overnight to allow attachment. For inflammation stimulation, the medium was replaced with the fresh one containing 1 μg/ml LPS. Different test samples were respectively added to the

inner well of the double-chamber system at 37 °C to explore their anti-inflammatory activities. Unstimulated RCK cultures incubated without SRCNs served as the Ctrl group. After a 2-day incubation, the release of IL-1β and TNF-α from RCKs into the conditioned medium was detected by the Quantikine ELISA kit (R&D Systems, Minneapolis, MN, USA). The presented results were averaged values from five independent runs.

1.5.4. In Vitro Anti-Angiogenic Activity Studies

Vascular endothelial growth factor (VEGF)-induced human umbilical vein endothelial cell (HUVEC) proliferation assay was first employed to evaluate anti-angiogenic properties of the SRCN samples. In a typical experiment, 5×10^3 HUVECs were seeded in a 48-well culture plate and incubated in endothelial cell growth (ECG) medium overnight for cell attachment. The ECG medium was then removed and the cells were washed with M199 culture medium (without FBS) for two times. Next, the HUVECs were incubated in M199 culture medium without FBS for 24 h to allow serum starvation. After that, the medium was removed and SRCNs in M199 culture medium (containing 1% FBS) and $VEGF_{165}$ (0.5 nM) were added to each well and a 24 h incubation was executed. Cell viability was assessed by PrestoBlue assay (n = 5).

Cell migration in ibidi Culture-Inserts was evaluated following the protocol by the manufacture. In short, 1.5×10^4 HUVECs were seeded in both wells of the Culture-Insert in a 24-well plate, and an overnight incubation in ECG medium was needed for cell attachment. Subsequently, the inserts were removed to produce a 180 μm cell-free gap. After a gentle wash with PBS, HUVECs were treated with VEGF-A_{165} (1 nM) in the absence (Ctrl) or presence of SRCNs in M199 medium containing 2% FBS. The wound size was quantified by ImageJ software and calculated as follow: migration distance (μm) = gap distance at 0 h – gap distance at 24 h (n = 5). In addition, the matrigel-based tube formation assay was carried out. To prepare matrigel-coated wells, 10 μl of matrigel matrix (10 mg/ml) was put in each well of a pre-chilled μ-slide angiogenesis chamber and incubated at 37 °C for gelation. HUVECs (2.5

× 10^4 cells/well) were further plated on the chamber slide and treated with VEGF-A_{165} (0.5 nM) for 24 h in the absence (Ctrl) or presence of SRCNs (10 μg/ml) in M199 containing FBS. Tube formation of the endothelial cells was assessed with an inverted photomicroscope (Olympus BX 51, Tokyo, Japan). Quantitative analysis of tubular structures was performed using ImageJ software, and total tube length was calculated as a percentage of that of the Ctrl (n = 5).

For the chicken chorioallantoic membrane (CAM) assay, fertilized chicken eggs were incubated at 37 °C for 4 days to obtain complete development of blood vessels and vasculogenesis. On day 5, after candling, two little holes were generated at two ends of the eggs and the CAM was separated from shell by a gentle force at one hole. The regions between the pre-existing vessels in the CAM were then exposed to sterilized cellulose paper discs (diameter of ~5 mm), which were pre-immersed in PBS (Ctrl) or a suspension of SRCNs in PBS. An Olympus digital camera was used to capture the CAM vasculature. The vascular density index was estimated by enumerating the number of intersections made by blood vessels with three equidistant concentric circles on the regions covered by discs (n = 5).

1.5.5. Bioactivity Mechanism of Action Studies

To inspect the influence of SRCNs on the signaling pathway mediating cell apoptosis, Western blot analysis was performed to identify p-IκB-α and NF-κB expression. After culturing for 12 h in a serum-free medium with or without SRCNs (1 mg/ml), RCKs were stimulated with 1 ng/ml IL-1β for 30 min at 37 °C. Unstimulated RCKs incubated without any substance worked as the Ctrl group. To prepare protein extracts, RCKs in each group were lysed in 1% NP-40 lysis buffer comprising 1 mM EDTA, 1 mM ethylene glycol tetraacetic acid, 5 μg/ml antipain, 5 μg/ml pepstatin A, 1 mM phenylmethylsulfonyl fluoride, and 5 μg/ml aprotinin.

Nuclear proteins for NF-κB Western blot analysis were then isolated using a nuclear extraction kit (Cayman, Ann Arbor, MI) following the manufacturer's instruction. Briefly,

cells were washed in 1 ml ice-cold PBS/phosphatase inhibitor solution, centrifuged at 300 × g for 5 min, redispersed in 500 ml ice-cold hypotonic buffer, placed on ice for 15 min, vortexed, and then centrifuged at 15000 × g for 30 s. The pelleted nuclei were mildly resuspended in 50 ml of ice-cold nuclear extraction buffer, vortexed for 15 s, and located on ice for 15 min; the nuclei were repeatedly suspended and incubated 6 times before the nucleus suspension was centrifuged at 15000 × g for 5 min at 4 °C. Aliquots of the supernatant containing soluble nuclear proteins were frozen in liquid nitrogen and kept at -70 °C.

Protein concentrations were assessed by a protein assay (Bio-Rad, Hercules, CA, USA). Subsequently, 50 μg protein per lane was separated by electrophoresis under reducing conditions in 10% polyacrylamide gel with sodium dodecyl sulfate (SDS-PAGE). For Western blotting, SDS-PAGE gels were transferred to poly(vinylidene difluoride) membranes, which had been blocked with 5% nonfat milk in tris-HCl-buffered saline containing 0.1% Twee-20 (TTBS) for 1 h at room temperature. Next, the membranes were incubated with the appropriate primary p-IκB-α and NF-κB antibodies (Cell Signaling, MA, USA) for overnight at 4 °C. After a 1-h incubation with anti-IgG-horseradish peroxidase (HRP), the secondary antibody, at room temperature for 1h, the blot was washed three times and developed using ECL Western Blotting Detection Reagents and an Analysis System Kit (GE Healthcare Amersham). Anti-β-actin (1:1000; Cell Signaling) was used as loading controls. The relative expression level of p-IκB-α and NF-κB was measured using ImageJ software. Values are mean ± SD (n = 4).

1.6. Functionalization of SRCNs and Cytocompatibility Studies

Before coating with poly(L-histidine), the SRCN samples were PEGylated. In particular, SRCNs and phosphonate-PEG-COOH (at 1:10 wt/wt ratio) were dispersed in DI water and the resultant solution was adjusted to pH 3. After removing excess molecules via an ultrafiltration process (30 kDa, Merck Milipore), the phosphonate-PEG-COOH modified SRCNs were dispersed in DI water. In this study, the weight ratio of phosphonate-PEG-

COOH to ceria fixed at 10:1 was based on our previous work,[2] where the functional polymer amount was optimized for the achievement of a uniform functional polymeric coating on the nanoparticle surface to further functionalize with poly(L-histidine). A higher ratio can result in a thicker phosphonate-PEG-COOH coating, and thereby poly(L-histidine) is simply conjugated to the exterior part of the thick phosphonate-PEG-COOH coating. Due to its weak physical interaction with the SRCN surface, the poly(L-histidine)/exterior phosphonate-PEG-COOH layer can be detached/corroded from the interior phosphonate-PEG-COOH/SRCNs during the processing (washing, dispersing, centrifuging, etc.). Next, the PEGylated SRCNs were dissolved in a MES buffer (5 ml) containing EDC (0.26 g) under agitation for 6 h, followed by mixing with poly(L-histidine) at different amounts [0.25 g (H1), 0.5 g (H2), and 1.0 g (H3)] in MES buffer (5 ml). The reaction was retained at room temperature for 24 h, and the resulting mixture was precipitated at 50 °C. To eliminate unbound poly(L-histidine) on the SRCNs, the precipitated solid was collected and washed with DI water three times, followed by re-dispersion in DI water. The dispersion was further centrifuged at 22000 × g for 10 min, and the precipitate was obtained as the product. Finally, the poly(L-histidine)-functionalized SRCNs were lyophilized at -50 °C and kept in a sealed vessel at room conditions.

The presence of functional groups and characteristic bands in the non-functionalized and functionalized SRCNs were verified by measuring their FTIR spectra in the range of 600–4000 cm^{-1} at an 8 cm^{-1} resolution with a FT-730 ATR/FTIR spectrophotometer (Horiba, Japan). Elemental composition of SRCNs and functionalized SRCNs were analyzed using an X-ray energy dispersive spectroscope (EDS) equipped in a JEOL JSM-1200EX II TEM system. Data acquisition was executed to quantify the nitrogen content (n = 5). Quantitative analysis of surface-bound amine groups on the functionalized SRCNs was conducted using the ninhydrin assay (n = 5). UV-vis absorption evolution from the SRCNs to functionalized SRCNs were examined using a UV-visible spectrophotometer (Thermo Scientific, Waltham, MA, USA). The zeta potential of various SRCN samples was measured via Doppler

microelectrophoresis (n = 5). DLS and FTIR measurements of SRCNs and functionalized SRCN samples were conducted similarly as the aforementioned.

1.7. Penetrating Capability Studies

To label the functionalized SRCNs with FITC, the nanomaterial sample was suspended in bicarbonate buffer solution (0.1 M Na_2CO_3, 0.1 M $NaHCO_3$, pH 9.5). Then, FITC (0.5 mg) was added to the suspension and stirred at room temperature for 24 h, followed by centrifugation. The amount of FITC in labeled samples was determined by monitoring the fluorescence intensity with excitation and emission wavelengths of 495 nm and 520 nm, respectively. The results were calculated with a standard curve of FTIC. It was found that the FITC contents in the SRCNs with different amounts of poly(L-histidine) are found to be at the same level.

To explore the permeability of functionalized SRCNs from epithelial cells to stromal cells, RCECs (1×10^5 cells) was seeded in a Transwell insert (0.4 μm pore, Corning). After 24 h, the seeded RCECs were treated with test materials. To study translocation of the functionalized SRCNs, the RCECs in the insert were co-incubated with RCKs in the lower chamber for 0.5 and 4 h. Next, the medium was sucked out, and the cells were washed with PBS. Cells in the lower chamber were stained with the DAPI for CLSM observation. The fluorescence reading was completed with the multimode microplate reader to detect the variance in the fluorescence intensity (n = 5). The permeability coefficient (P) was calculated as follows: $P = Q/Act$, where Q/t is the total amount of the functionalized SRCNs transported into the receiver compartment per unit time, A the diffusion area of the cell layers, and c the initial concentration of the functionalized SRCNs in the donor compartment. Results were averaged from five independent measurements.

1.8. In Vitro Drug Release Studies

For the assessment of drug loading content and entrapment efficiency, functionalized SRCN dispersions (1 mg/ml) were mixed with ACh or SB431542, followed by adding with

BSS (1.5 ml) at a controlled pH 6.0 in a vial. The resulting mixture was then sonicated for 2 h and further stirred for 24 h at room temperature. Next, the pH value of the mixture was adjusted to 7.4, and the mixture was stirred for 12 h. Subsequently, the drug-loaded SRCNs were separated by centrifugation and washed thoroughly with BSS (pH 7.4) to eliminate drug molecules absorbed on exterior surfaces of the functionalized SRCNs. The amount of free drugs in the decanted supernatants was quantified using a high-performance liquid chromatography (HPLC) system containing a L-2400 UV detector (Hitachi, Tokyo, Japan) and a Mighty RP-18 column (4.6 × 250.0 mm; Kanto Chemical, Tokyo, Japan). To achieve the drug release profiles, 3.0 mg of each drug-loaded nanocarriers was dispersed in BSS (5 ml) at a controlled pH 6.0 and 7.4, respectively. Release buffers were collected at predetermined time points and the drug amounts were analyzed by the HPLC (n = 4). To explain possible mechanisms of the drug released from the nanocarriers, drug release kinetics were assessed using the Higuchi equation: $Q = kt^{1/2}$, where Q is the amount of drug released at time t and k is the release constant.

1.9. Pharmacological Activity Studies

To evaluate pharmacological activities of ACh released from the functionalized SRCNs, RCKs (5×10^4 cells/ml) were seeded into 24-well plates by 1 ml/well and incubated with 20 µl of various drug-loaded nanocarriers (H0: 20 µg Ce + 0.66 µg ACh; H1: 20 µg Ce + 5 µg H + 2.70 µg ACh; H2: 20 µg Ce + 10 µg H + 4.70 µg ACh; H3: 20 µg Ce + 20 µg H + 6.12 µg ACh) for 2 days; cells treated without drug-containing materials referred to Ctrl groups. The metabolic activity was evaluated by a cell proliferation MTS assay. The microplate spectrophotometer was employed to determine the absorbance at 490 nm. Results were presented as relative MTS activities to that of the Ctrl (n = 4). For cell cycle analysis, RCKs (1×10^5 cells/ml) were seeded into 6-well plates at 1 ml/well and stained with propidium iodide. After a 2-day co-culture with the drug-loaded nanocarriers, RCKs were trypsinized and resuspended in PBS at 1×10^6 cells/ml, followed by fixing with cold ethanol (70%) for

overnight at -20 °C. The fixed cells were then incubated with propidium iodide staining solution containing 100 μg/ml propidium iodide, 20 μg mg/ml RNase A, and 0.1% Triton X-100 for 30 min at room temperature. The percentages of cells in the G0/G1, S, and G2/M phases were analyzed using flow cytometry. The extracellular matrix production levels in culture supernatants and lysed cells were quantitated for total glycosaminoglycans (GAG) and collagen contents. The amount of hydroxyproline, an amino acid marker for collagen, was determined using the protocol for quantification of hydroxyproline in aqueous humor with slight modifications. Briefly, after hydrolyzed with 6 N HCl for 18 h at 110 °C, the samples (supernatants and lysed cells) were mixed with a buffered chloramine-T reagent, and the oxidation reaction was allowed for 25 min at room temperature. After adding Ehrlich's aldehyde reagent to the sample, the absorbance was read at 550 nm by using a spectrophotometer (ThermoLabsystems, Vantaa, Finland) and correlated with a standard calibration curve to obtain the hydroxyproline amount. All experiments were carried out in quadruplicate.

The total GAG content was determined by a colorimetric assay using dimethylmethylene blue (DMMB) reagent. In short, the culture supernatants and papain-digested cells were mixed with DMMB reagent solution (40 mM NaCl; 40 mM glycine; 46 μM DMMB, pH 3.0). The absorbance was read at 525 nm by the spectrophotometer and compared with a standard curve of chondroitin sulfate A. All experiments were conducted in quadruplicate.

To assess the pharmacological activity of SB431542 released from the functionalized SRCNs, an enzyme-linked immunosorbent assay (ELISA) was employed to examine anti-fibrotic activities. In particular, RCKs (5×10^4 cells/well) were seeded in 24-well plates containing regular growth medium and incubated overnight. For transforming growth factor (TGF)-β1 stimulation, the medium was substituted by the fresh one containing 2 μg/ml TGF-β1. Each well of the 24-well plate was divided into two compartments by cell culture inserts (Becton Dickinson Labware). Different test samples were separately added to the inner

compartment at 37 °C to assess their anti-fibrotic activities on the TGF-β1-stimulated culture; unstimulated RCKs incubated with no test substances worked as the Ctrl groups. After a 2-day incubation, the release of α-smooth muscle actin (α-SMA) and TGF-β1 from untreated/treated RCKs into the conditioned medium was recognized by the Quantikine ELISA kit (R&D Systems, Minneapolis, MN, USA). The protein levels of p-Smad2 and Smad2 in RCKs was determined by Western blotting. To prepare protein extracts, cells were lysed in 1% NP-40 lysis buffer containing 1 mM EDTA, 1 mM ethylene glycol tetraacetic acid, 5 μg/ml antipain, 5 μg/ml pepstatin A, 1 mM phenylmethylsulfonyl fluoride, and 5 μg/ml aprotinin. Quantification of protein concentrations were performed the protein assay (mentioned earlier), and 50 μg of protein per lane was separated by electrophoresis under reducing conditions in 10% polyacrylamide gel with SDS-PAGE. The SDS-PAGE gels were moved to poly(vinylidene difluoride) membranes, blocked with 5% nonfat milk in tris-HCl-buffered saline containing 0.1% Twee-20 (TTBS) for 1 h at room temperature, for Western blotting. Subsequently, the membranes were incubated with primary antibody at 4 °C for overnight. The blots were further incubated with a secondary antibody at room temperature for 1 h. SuperSignal West Pico chemiluminescent substrate (Pierce, Rockford, IL, USA) was utilized for detection of the secondary antibody on imaging films (Biomax MS, Eastman Kodak, Rochester, NY, USA). The protein bands were analyzed by densitometry using ImageJ software. Data were averaged from four independent experiments.

Sprage-Dawley rats were purchased from the BioLASCO Experimental Animal Center (Taipei, Taiwan, ROC). To assess the concentration of nanocarrier/drug materials in the stroma, the rats inflicted with corneal alkali burn (AB) received topical instillation of 20 μl of materials including H0 (20 μg Ce, 0.66 μg ACh, and 0.14 μg SB), H1 (20 μg Ce, 5 μg H, 2.70 μg ACh, and 1.50 μg SB), H2 (20 μg Ce, 10 μg H, 4.70 μg ACh, and 3.36 μg SB), and H3 (20 μg Ce, 20 μg H, 6.12 μg ACh, and 4.66 μg SB). Rats were sacrificed at 4 days post-administration, and rat corneas were isolated and homogenized in 100 μl of sterile PBS,

followed by centrifugation at 22000 × g for 20 min at 4 °C. Finally, the supernatant was collected and stored at -80 °C until further analyses. The remaining Ce content in the rat corneal tissues was analyzed by the ICP-MS (*n* = 4). The drug concentration resided in the rat corneas was determined using HPLC (*n* = 4).

1.10. In Vivo Biocompatibility Studies

Following topical instillation of test samples to the rats, the morphology of ocular anterior segment was observed using a slit-lamp biomicroscope (Topcon Optical, Tokyo, Japan). The rat corneas were excised and processed for histological examinations after the animals were euthanized by CO_2 gas. The corneal tissues were fixed in 4% paraformaldehyde in PBS, followed by dehydration in ethanol solutions, embedment in paraffin, and slice into 5 μm-thick sections. These sections were stained with hematoxylin and eosin (H&E) and visualized using a light microscope (Carl Zeiss).

1.11. In Vivo Therapeutic Studies

Corneal haziness was assessed using the slit-lamp biomicroscope at 8 h and 4 d post-instillation, respectively. Results were classified according to a previous study.[3] Briefly, grade 0, completely clear, no opacity can be observed by any methods of slit-lamp microscopic examination; grade 0.5, a trace or a weak haziness that can be seen only by indirect broad tangential illumination; grade 1, haze with a minimal density that can hardly be seen with direct and diffuse focal slit illumination; grade 2, more prominent haze easily visible with mild obscuration of iris; grade 3, a moderately dense haziness that partially obscures the iris; and grade 4, a severely dense opacity that obscures completely the details of intraocular structure. Corneal epithelial defects were examined by staining the test corneal surfaces with fluorescein solution at 8 h and 4 d post-injury. The area ratio of the green stained areas to the entire cornea was calculated using ImageJ software. The percentage of corneal wound area was calculated as follows: remaining area of staining after treatment/initial wound area × 100%. Corneal angiogenesis was assessed using the slit-lamp

biomicroscope at 8 h and 4 d post-treatment, respectively. Results were classified according to a previous report.[4] An ultrasound biomicroscope (Accutome 4Sight UBM module, Keeler) was employed to assess the corneal thickness of test eyes in dark using a 48-MHz transducer. Radial scans were conducted in different quadrants of the angle. One scan per quadrant of each test eye was recorded for the analysis; The central corneal thickness was quantified by UBM imaging (n = 10). Corneal topographic maps were obtained using a Medmont E300 Corneal Topographer (Medmont Pty Ltd., Melbourne, Australia). The corneal topographer was calibrated following the manufacturer's instruction (± 0.01 mm at four calibration surfaces). The mean keratometric (K) value, the average corneal curvature in the 3-mm central zone, was achieved from the topographic map (n = 10).

1.12. Therapeutic Activity Studies

1.12.1. In Vivo Anti-Inflammatory Activity Studies

Rat eyes were immediately frozen in an optimal cutting temperature (OCT) compound after removal from the scarified animals at 4 d post-treatment. The OCT-embedded corneal sections were prepared with a thickness of 5 μm for immunofluorescence staining. These tissue sections were then fixed in ice-cold acetone for 20 min and washed in PBS. After blocking in 1% BSA, immunofluorescence staining was conducted with the following primary antibodies rat anti-CD11b (1:10; Hybridoma Bank, Iowa City, IA, USA) as well as the secondary antibodies Alexa Fluor® 594 donkey anti-rat IgG (H+L) (1:200; Invitrogen). After washing with tris-buffered saline with 0.1% Tween® 20 detergent for three times, the sections were further incubated with mounting medium with the DAPI. Fluorescence signals were acquired using the fluorescence microscope.

For real-time reverse transcription polymerase chain reaction (RT-PCR), the corneal tissues collected from the enucleated eyes were immediately placed into RNAlater solution (Life Technologies, Carlsbad, CA, USA) and stored at -80 °C until analysis. The Qiagen

RNeasy Mini Kit (Qiagen, Hilden, Germany) was used to extract total RNA from the corneal tissues to quantify the mRNA expression levels of IL-1β and TNF-α.

1.12.2. In Vivo Anti-Oxidant Activity Studies

At 4 d post-treatment, the entire corneas from the test animals were excised and fixed with 4% paraformaldehyde for 1 d. Next, the corneas were removed, embedded, and processed into 5 μm thick slices. Unfixed cryosections (10 μm) were incubated with 100 μM DCFH-DA or 5 μM dihydroethidium (DHE, Sigma-Aldrich, St. Louis, MO, USA) at 37 °C for 15 min. After washing with PBS three times, the sections were further incubated with mounting medium with the DAPI. Fluorescent signals were recorded under the fluorescence microscope.

Biochemical assays including superoxide dismutase (SOD), catalase (CAT), and malondialdehyde (MDA) levels were used to assess the activity of the antioxidant defense system in the eyes inflicted with the chemical injury. AB rats were randomly divided into six groups (n = 10). At day 4 post-treatment, rats were sacrificed to harvest the entire corneal tissues for biochemical assays following the protocol of the kit manufacturer.

1.12.3. In Vivo Anti-Apoptotic Activity Studies

Apoptosis of corneal cells in the tissue sections was examined by TUNEL assay (Roche Diagnostics). After fixing with 4% paraformaldehyde, the corneal tissues were permeabilized in 0.1% Triton X-100 in 0.1% sodium citrate for 2 min on ice, and then incubated with a mixture of TdT solution and fluorescein isothiocyanate dUTP solution in a humidified chamber for 1 h at 37 °C. The negative controls were incubated with distilled water as a substitute for the TdT enzyme. The tissue sections were counterstained with the DAPI and cell nuclei were visualized under the fluorescence microscope. Three random areas were chosen, and the number of TUNEL-positive apoptotic cell nuclei was quantified accordingly.

1.12.4. In Vivo Anti-Angiogenic Activity Studies

Three relaxing radial incisions from corneal tissues of the test eyes were made at day 4 post-treatment. The corneal flat mounts were rinsed in PBS, fixed in acetone, and rinsed in PBS again. Subsequently, the mounts were blocked in 10% normal donkey serum (Shanghai Yeasen Biotechnology Co., Ltd., Shanghai, China), stained with rat anti-mouse CD31 (1:150; BD Biosciences) for overnight at 4 °C, and washed with PBS. After that, the specimens were incubated with FITC for 1 h at room temperature in dark conditions. Digital images of the flat mounts were photographically captured, and the area stained with CD31 was measured morphometrically using ImageJ software. The total area of neovascularization was normalized to the whole corneal area, and the percentage of the cornea covered by blood vessels was calculated accordingly (n = 10).

Test eyes were enucleated, formalin-fixed, and sectioned at 4 µm. The sections were then deparaffinized and incubated at 95 °C for 25 min in 0.1 M citrate (pH 6.0) for antigen retrieval and use in immunohistochemical studies. The primary antibodies were used anti-CD31 (10 pg/ml; Dianova, Hamburg, Germany), and immunostaining detection was performed using anti-rat IgG following instructions from the manufacturer. Sections were counterstained and mounted. Bound anti-CD31 was detected using Alexa Fluor® 594 donkey anti-rat IgG (H+L) (1:200; Invitrogen) and immunofluorescence evaluation. Sections were counterstained with the DAPI. Images were obtained at 400× magnification by bright-field microscopy. Quantitation of CD31 fluorescent staining was performed using ImageJ software.

The corneas harvested from the test eyes were dissected and frozen at -70 °C, followed by homogenization in ice-cold RIPA lysis buffer solution (Santa Cruz Biotechnology Inc., Santa Cruz, CA, USA). After a 5 min-centrifugation at 12000 × g, the supernatants were decanted and the protein concentrations were estimated using the Bradford reagent (Sigma-Aldrich Chemie GmbH, Steinheim, Germany). Equal quantities of protein (15 µg/lane) from the cell lysates were separated using 8–15% SDS-PAGE and moved onto nitrocellulose membranes (Santa Cruz Biotechnology, Inc.). These membranes were then incubated for 2 h

in PBS plus 0.1% Tween-20 and 5% non-fat skim milk for blocking non-specific binding. The membranes were further incubated overnight at 4 °C with goat monoclonal anti-mouse VEGF (1:2000; Santa Cruz Biotechnology, Inc.) and anti-β-actin (1:1000; Cell Signaling). After incubating with the HRP secondary antibody at room temperature for 1 h, the blot was washed three times and developed using ECL. The chemiluminescent substrate was then employed to detect the secondary antibody on the imaging films. The protein bands were analyzed by densitometry using ImageJ software. Results were averaged from ten independent runs.

1.13. Tissue Structure/Function Analysis

The gross appearance of excised corneal tissues from the test eyes was imaged with a digital camera (Nikon, Tokyo, Japan). For histological study, the rat corneas were excised and fixed in 4% paraformaldehyde in PBS, followed by dehydration in a grade series of ethanol solutions, embedment in paraffin, and slice into 5 μm-thick sections. After that these corneal sections were stained with H&E and immunohistochemistry, and examined under the light microscope for estimation of the total corneal thickness (n = 10). Masson's trichrome were performed as follows: the slides were first deparaffinized in xylene, then the corneal stromal tissues were fixed on the slides with graded alcohol and stained with titrate Plumbine's trichrome; in each step, 1% acetic acid was used as a washing agent.

For TEM examination, corneal stromal specimens were dehydrated in ethanol and embedded in resin. The tissue specimens were then sectioned, located on a copper grid, and stained with uranyl acetate. Young's moduli were measured using a universal testing machine (Instron, Canton, MA, USA) at a crosshead speed of 0.5 mm/min under ambient conditions until a rupture of the corneal tissues (n = 10).

For immunohistochemical staining, corneal sections were fixed in cold acetone for 5 min and were processed using the anti-α-SMA (1:100; Abcam, Cambridge, MA, USA). The corneas were dissected and frozen at -70 °C, then homogenized in the ice-cold RIPA lysis

buffer solution. Following centrifugation at 12000 × g for 5 min, the supernatants were collected and the protein concentrations were estimated using the Bradford reagent. Equal quantities of protein (15 µg/lane) from the cell lysates were separated using 8–15% SDS-PAGE and located on nitrocellulose membranes, which were further incubated in PBS containing 0.1% Tween-20 and 5% non-fat skim milk for 2 h to block non-specific binding. Subsequently, the membranes were then incubated overnight at 4 °C with anti-α-SMA (1:100; Abcam), TGF-β1 (1:200; Abcam), and GAPDH (1:1000; Abcam). After incubating with the HRP secondary antibody at room temperature for 1 h, the blot was washed three times and developed using ECL. The chemiluminescent substrate was used for detection of the secondary antibody on the imaging films. Analysis of the protein bands were conducted by densitometry using ImageJ software. Results were averaged from ten independent runs. To measure the cerium concentration in vivo, the rats were euthanized with CO_2 gas at different follow-up time points (8 h, 4 d, and 10 d). The aqueous humor from the rat eyes were immediately aspirated using 30-gauge needles. Additionally, rat corneas were harvested and homogenized in sterile PBS, followed by a centrifugation (15000 rpm) for 20 min at 4 °C. The supernatants were further collected, and cerium retention in the corneal tissue and aqueous humor specimens was analyzed by the ICP-MS (n = 10).

2. Supplemental Results and Discussion

2.1. Bioactive Propertiey

It is acknowledged that ceria NPs are multifunctional nanomaterials that can be exploited not only as therapeutic agents, but also as bioactive nanocarriers for synergistically boosting the treatment efficacy of various drugs. Here, the bioactive characteristics including anti-oxidant, anti-apoptotic, anti-inflammatory, and anti-angiogenic properties of SRCNs were investigated *in vitro* with respect to different degrees of surface roughness to rationally achieve the intrinsically therapeutic nanocarriers with highest performances.

Given that oxidative stress causes the influx of calcium ions into the cytoplasm and an overload of calcium ions can prompt cell death,(5) intracellular calcium levels in RCKs treated with SRCNs and hydrogen peroxide were also examined. **Figure S5**a and b indicated that as the surface roughness augmented, the calcium signal (blue fluorescence intensity) reduced, i.e., rougher SRCNs could reduce calcium overload in the RCKs at greater extents.

2.2. Nanocarrier Functionalization

To enhance cell/tissue penetration capability of the SRCNs, PEGylation was first employed to endow the nanocarriers with COOH-surface functional groups, thereby providing active sites for grafting with poly(L-histidine) via amide bond formation. Considering that the COOH moieties can be reacted with amino groups of poly(L-histidine), different amounts of the polypeptide are employed to rationally obtain the surface functionality while minimizing possible alterations in morphological structures of SRCNs. The representative TEM images (**Figure 3**a) of the PEGylated and poly(L-histidine)-functionalized SRCNs and their corresponding elemental EDS maps confirmed that the chemical modification/functionalization exerted no obvious impact on the ceria nanocage structures while introducing new chemical elements (P and N). Quantitative chemical analyses (**Figure 3**b and c) revealed that the N element and amino group contents on the SRCNs increased with increasing the polypeptide amounts during the carbodiimide chemistry for the attachment of poly(L-histidine) to COOH-terminated surface; noting that the polypeptide feeding amount to obtain H3 specimen was sufficient enough for consuming most COOH moieties on the PEGylated-SRCNs, as verified by the grafting yield close to 100% (**Figure 3**c). These results were further supported by the gradual evolution of zeta potentials from negative to positive (**Figure 3**d), as poly(L-histidine) is positively charged.

The FTIR spectra (**Figure S6**a) indicated the presence of a new peak at 1639 cm^{-1} (amide I band) in the functionalized SRCNs (H1, H2, and H3) and the gradual increase in intensity of

this peak with increasing the polypeptide coating amounts, signifying successful functionalization of different poly(L-histidine) amounts on the nanocarriers. On the other hand, the physical measurements (**Figure S6** b–d) indicated that all the PEGylated/functionalized SRCNs (H0, H1, H2, and H3) possessed comparable size distributions and diameters, exhibiting a good accordance with the TEM observation (**Figure 3**a). It is important noting that the surface roughness of the SRCNs is not apparently affected by the chemical modification and functionalization (**Figure S6**e and f), possibly due to the relatively low molecular weights (i.e., small sizes) of the functional biopolymers and the effective removal of unbound residues from the nanocarrier surfaces. In addition, the surface coatings were demonstrated to be harmless to ocular biocompatibility of SRCNs *in vitro* (**Figure S7**), possibly due to the good biocompatible nature of poly(L-histidine).

2.3. In Vitro Pharmacological Activities

MTS assays indicated the metabolic activities (**Figure 4**c) of the corneal cells were higher in all nanoformulation groups and steadily increased with increasing the polypeptide coating amounts. This result infers the effectiveness of the functional polypeptide in improving the *in vitro* pharmacological activity of the released ACh. Flow cytometric analysis (**Figure S8**e and **Figure 4**d) further confirmed that the released ACh was capable of promoting cell proliferative capacity and the RCK growth was proportional to the polypeptide amount on the SRCNs, as evidenced by more RCKs at the S phase fraction of the cell cycle. To support these, quantitative analysis of GAG and collagen (major matrix components of the corneal stroma) secreted from different RCK cultures was performed. Both total GAG (**Figure 4**e) and collagen (**Figure S8**f) contents showed a strong correlation with the cell proliferation data, implying a potential strategy for improving corneal wound repair of ACh by rationally designing an effective/increased release from the poly(L-histidine)-functionalized SRCNs.

To evaluate whether the functionalized SRCNs co-loading with ACh/SB431542 could be employed as the ophthalmic nanoformulations, the bioavailability of ceria (the therapeutic component of the functionalized SRCNs) and the two drugs in rat corneas inflicted with chemical injury was analyzed (**Figure 4**i and j). The results showed that the amounts of Ce, ACh, and SB431542 were significantly improved with increasing the polypeptide functionalization level on the SRCNs. On the other hand, to validate the pH-responsive effect of nanoformulations, we performed comparison experiments by topical instillation of the drug-loaded SRCNs to healthy rat eyes (neutral environment). The results showed a relatively low drug level in cornea (**Figure S8**i), which is consistent with the findings of in vitro drug release tests (**Figure 4**a, b), further verifying the pH-responsive performance of nanocarriers. Taken together, our data demonstrate the critical roles of poly(L-histidine) coatings in facilitating transcellular transport of the drug-containing SRCNs through the corneal epithelium to the injured/inflamed corneal stroma where the functional biopolymer coatings further regulate the release of the medications in response to the pH change; it is worth noting that the bioavailability of the therapeutic nanocarriers and drugs is proportional to the polypeptide coating amounts (**Figure S9**).

2.4. Pharmacotherapy of Corneal Burn

Before pre-clinical studies on the diseased eyes, biocompatibility of our developed nanoformulation (denoted as ACh+SB/Ce-H) was assessed. Slit-lamp biomicroscopy and corneal fluorescein staining examination (**Figure S10**a and b) of healthy eyes respectively receiving topical instillation of ATS and ACh+SB/Ce-H and its components including SRCNs (Ce) and poly(L-histidine)-functionalized SRCNs (Ce-H) revealed no obvious difference at 4 days post-administration; all groups exhibited clear cornea and quiet anterior chamber as well as no fluorescein stain absorbed on the ocular surface, signifying that the topical ophthalmic nanoformulation was harmless to the eye. Furthermore, histological H&E staining images

(**Figure S10**c) of the treated healthy corneas in all groups showed a multilayered tissue structure comprising the neat epithelium (top layer) without any abnormalities, demonstrating the high biocompatibility of the developed nanoformulation. Pharmacological treatment efficacy of our ACh+SB/Ce-H nanoformulation was then evaluated via comparative studies with a commercial dexamethasone eye drop.

In addition, ocular surface abnormalities of the AB eyes in response to different treatments were examined using fluorescein staining (**Figure 5**b and **Figure S10**e). The occupation of the green stain on the whole corneal region of the AB eyes signified the development of injury-induced defects fully distributed on the ocular surface. The areas of the corneal injury were gradually decreased in all test groups. The slight reduction of corneal wound areas in the Ctrl group can be ascribed to the epithelial layer, which is a self-renewing tissue with capabilities of providing a long-lasting source of proliferating cells for epithelial regeneration.[(6)] Our nanoformulation and its components all outperformed Dex in promoting corneal wound healing after the injury; specifically, the ACh+SB/Ce-H revealed the highest performance with a 19-fold improvement in the reduction of corneal wound areas as compared to the marketed eye drops. This improved treatment efficacy can be attributed to the effective pharmacological activity of ACh, which is enabled by the functionalized SRCNs. Furthermore, considering that ocular surface neovascularization is a noticeable feature in chemical eye injuries, slit-lamp biomicroscopy was utilized to observe possible inhibitory effects of the nanoformulation on the abnormal blood vessels (**Figure 5**c and **Figure S10**f). The formation of vascular network could be observed in the AB group, and the vascularization was rapidly progressed in the Ctrl group during the follow up (i.e., ATS or the diseased eyes possessed no inhibitory effects). Dex or Ce could only suppress the abnormal blood vessels in a short time (e.g., 8 h) and their pharmacological activities were totally lost at 4 days post-instillation, as proved by the reoccurrence of even denser vascular networks. Interestingly, both Ce-H and ACh+SB/Ce-H exhibited equal treatment efficacies in

attenuating abnormal blood vessels at the strongest level (~93% of the abnormal blood vessels were suppressed, as compared to AB group), possibly due to the potent anti-angiogenic properties of the ceria component.[7] Although consisting of comparable ceria amounts, the low treatment efficacy in the Ce group could be referred to the poor cell/tissue penetration capability of the ceria-based nanocarriers without the poly(L-histidine) coating.

Chemical corneal injuries can trigger a complex wound healing process, including transdifferentiation of keratocytes into myofibroblasts. Noting that myofibroblasts are highly contractile cells capable of secreting and depositing great amounts of matrix components in the stromal tissue, consequently increasing the corneal thickness;[8,9] moreover, inflammation progression is another factor that causes corneal edema.[10] In this regard, ultrasound biomicroscopy (**Figure 5**d and **Figure S10**g) and corneal topography (**Figure 5**e and **Figure S10**h) measurements were conducted to determine whether the pharmacological treatments could exert an effect on the corneal thickness/structure. The results indicated that at 8 h post-administration, except the Ctrl, all groups exhibited a reduction in both corneal thickness and curvature from severe states (i.e., high thickness and large curve in AB eyes). Nevertheless, this decreasing trend only maintained in the Ce-H and ACh+SB/Ce-H groups as the follow-up was extended to day 4. In particular, the corneal thickness and curvature in the ACh+SB/Ce-H could be restored to normal conditions compared to those in Pre group. The effectiveness of Ce-H can be ascribed to the potent anti-inflammatory activities of ceria component in reducing inflammation-mediated corneal edema. The superlative treatment efficacy of ACh+SB/Ce-H can be explained due to its capability of delivering SB431542 (in addition to other medications) into the corneal tissue, consequently triggering anti-fibrotic activities of the drug in the corneal tissues to restore structural changes in the corneas caused by the chemical injury.

References

(1) L. J. Luo, D. D. Nguyen, J. Y. Lai, *Theranostics*, **2021**, *11*, 5447–5463.

(2) L. J. Luo, D. D. Nguyen, J. Y. Lai, *Biomaterials*, **2020**, *243*, 119961.

(3) F. E. Fantes, K. D. Hanna, G. O. Waring, Y. Pouliquen, K. P. Thompson, M. Savoldelli, *Arch Ophthalmol.* **1990**, *108*, 665–675.

(4) T. S. Jiang, L. Cai, W. Y. Ji, Y. N. Hui, Y. S. Wang, D. Hu, J. Zhu, *Mol. Vis.* **2010**, *16*, 1304–1316.

(5) M. Zhang, R. Song, Y. Liu, Z. Yi, X. Meng, J. Zhang, Z. Tang, Z. Yao, Y. Liu, X. Liu, W. Bu, *Chem* **2019**, *5*, 2171–2182.

(6) A. V. Ljubimov, M. Saghizadeh, *Prog. Retin. Eye Res.* **2015**, *49*, 17–45.

(7) J. M. Yong, L. Fu, F. Tang, P. Yu, R. P. Kuchel, J. M. Whitelock, M. S. Lord, *ACS Biomater. Sci. Eng.* **2022**, *8*, 512–525.

(8) S. E. Wilson, *Exp. Eye Res.* **2020**, *201*, 108272.

(9) J. Luisi, E. R. Kraft, S. A. Giannos, K. Patel, M. E. Schmitz-Brown, V. Reffatto, K. H. Merkley, P. K. Gupta, *Transl. Vis. Sci. Technol.* **2021**, *10*, 6.

(10) D. G. Goswami, N. Mishra, R. Kant, C. Agarwal, C. R. Croutch, R. W. Enzenauer, M. J. Petrash, N. Tewari-Singh, R. Agarwal, *PLoS One* **2021**, *16*, e0258503.

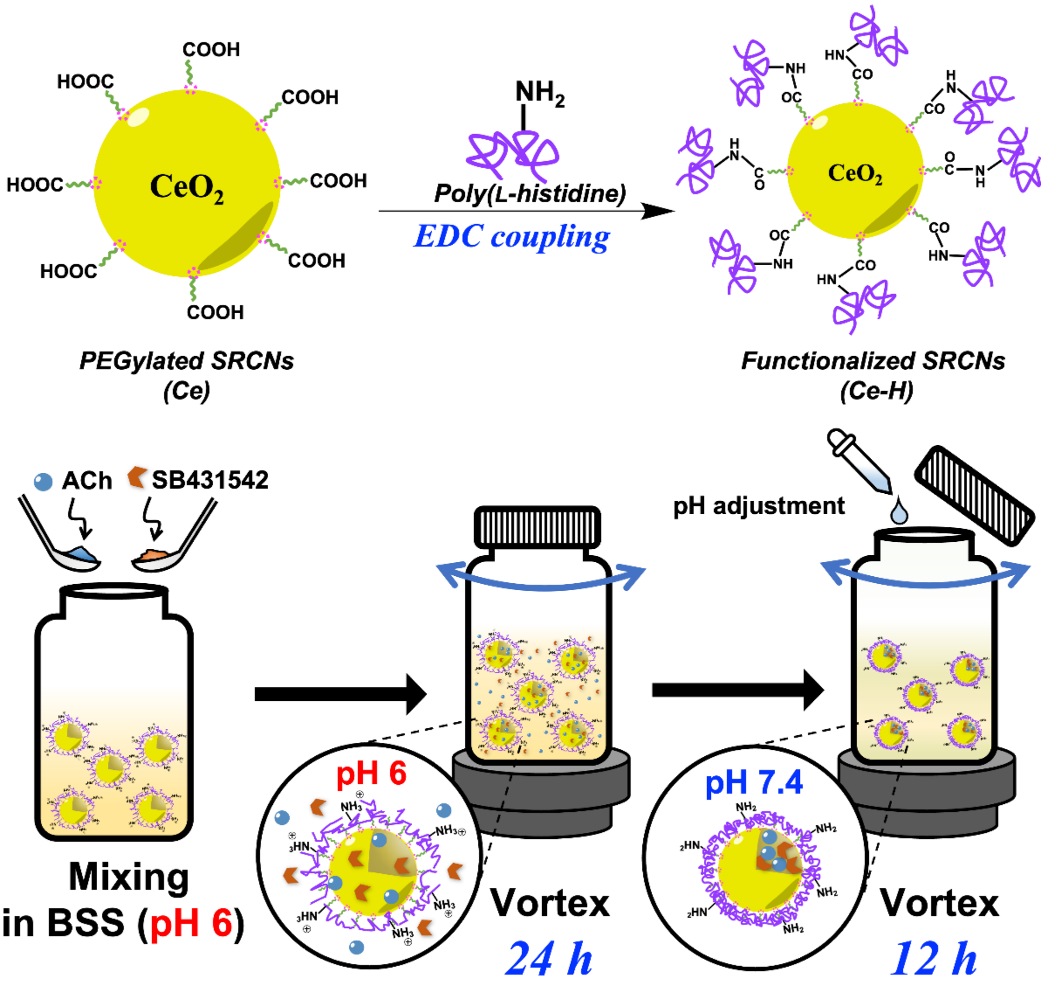

Figure S1. Schematic illustration for the development of the SRCN-based ophthalmic nanoformulation.

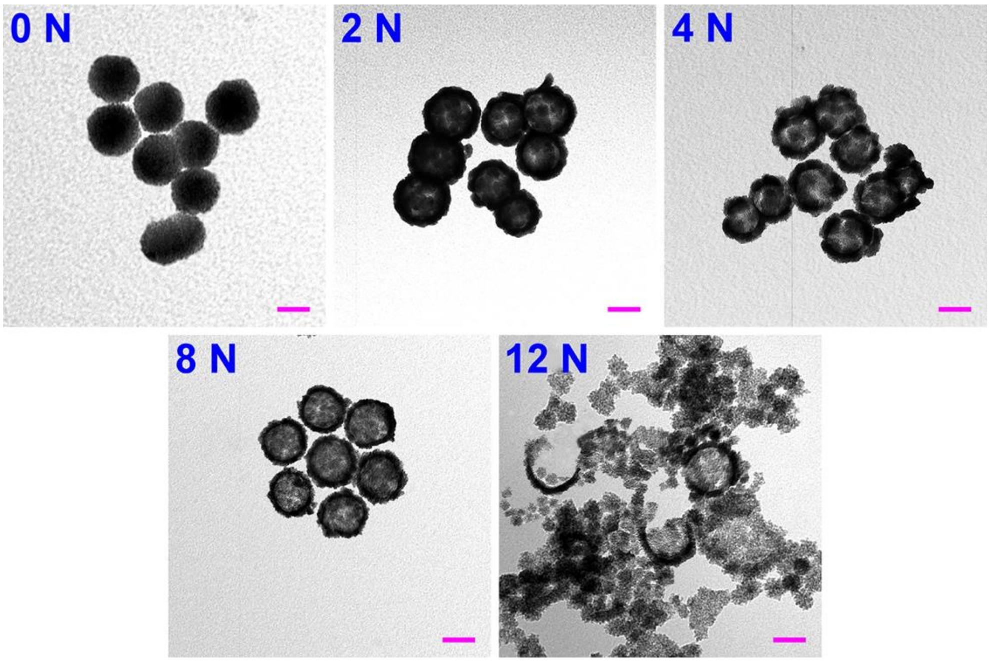

Figure S2. TEM images of SRCN samples fabricated using different NaOH concentrations (0 N, 2 N, 4 N, 8 N and 12 N). Scale bars are 50 nm.

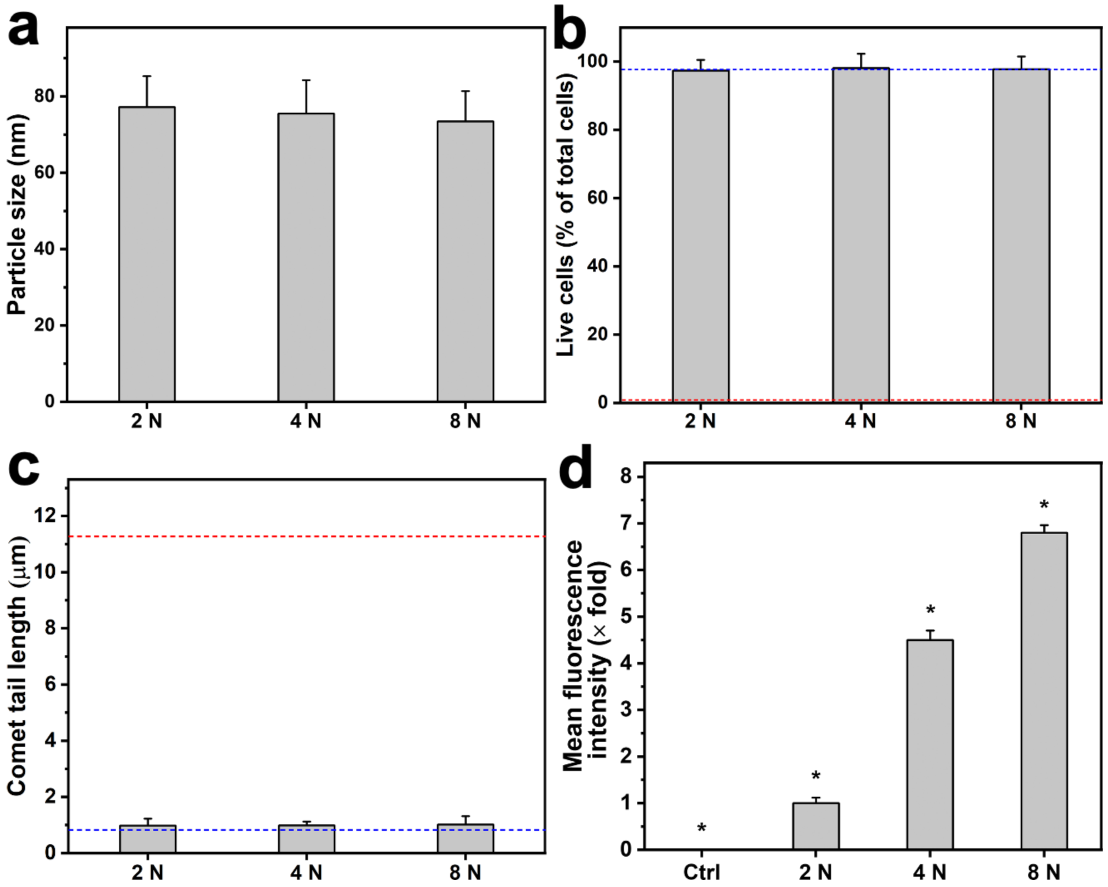

Figure S3. (a) Particle diameters of the various SRCN samples measured by DLS (n = 5). (b) Mean percentage of live cells in the RCK cultures exposed to different SRCN samples (2 N, 4 N and 8 N) determined by the live/dead assay; the blue and red dash lines represent the values of Ctrl and MetOH group, respectively. Values are mean ± SD (n = 4). (c) Comet tail length of RCKs exposed to various SRCN samples; the blue and red dash lines represent the values of Ctrl and Cd group, respectively. Values are mean ± SD (n = 4). (d) Mean fluorescence intensity of various types of FITC-labeled SRCNs. Values are mean ± SD (n = 5); *P < 0.05 vs all groups.

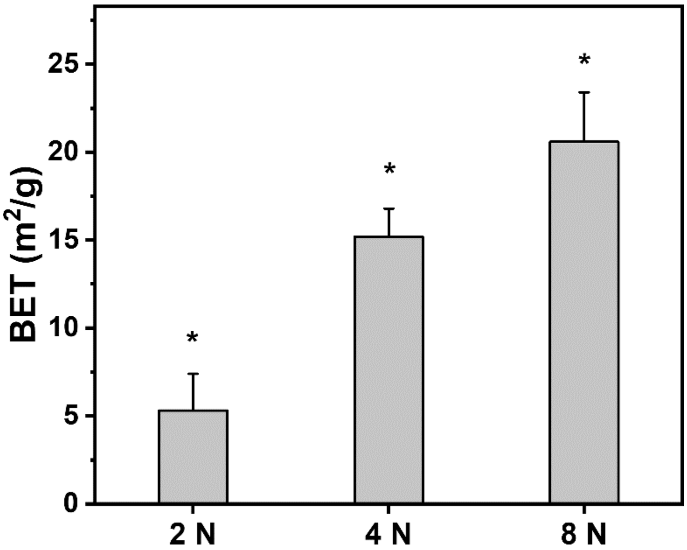

Figure S4. BET surface areas of the SRCNs fabricated using different etchant concentrations. Values are mean ± SD (*n* = 5); *$P < 0.05$ vs all groups.

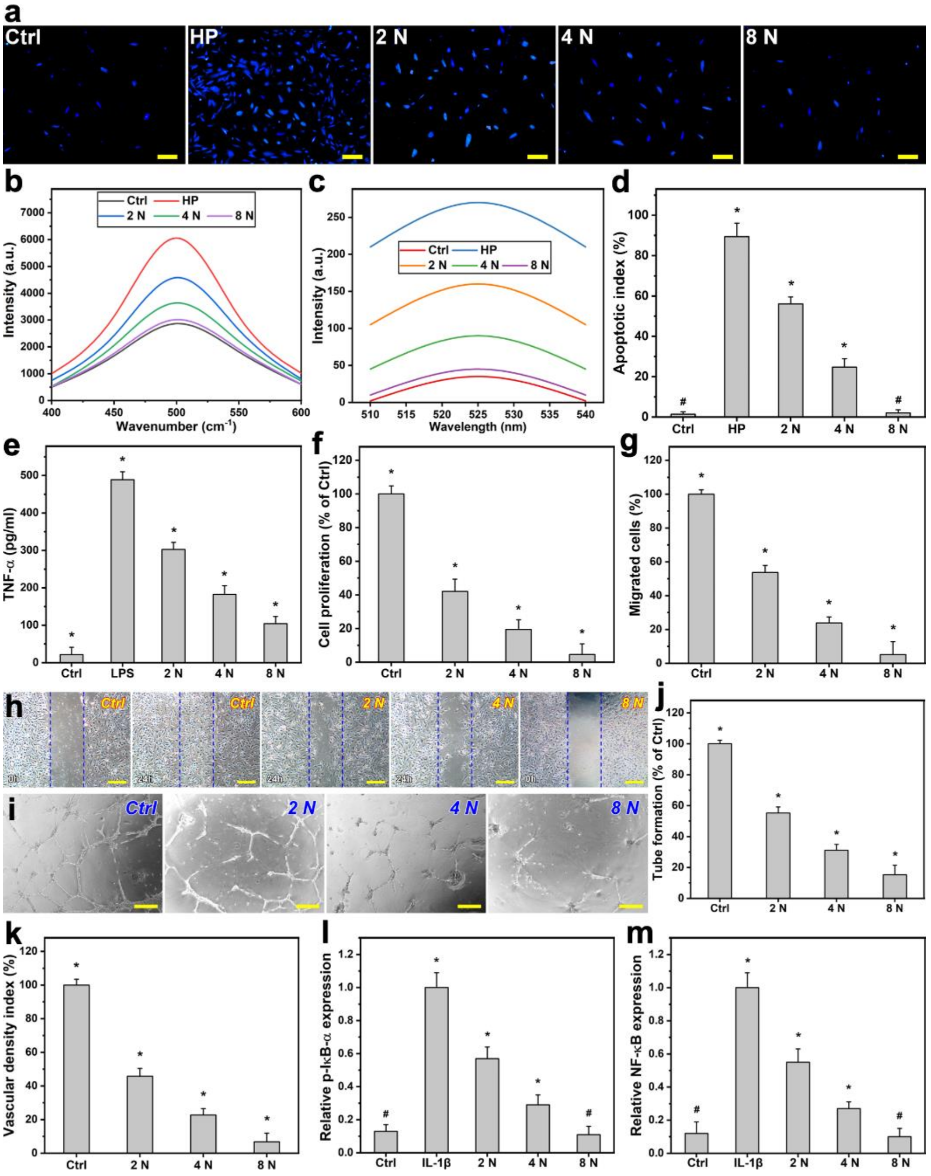

Figure S5. (a) Representative images of the Fura-2 AM-labelled RCKs after incubation with various types of SRCNs for 24 h and further exposure to H_2O_2 for 24 h. The cells exposed to 0 (Ctrl group) or 200 (HP group) μM H_2O_2 for 24 h without 24 h of pretreatment with any materials were used for comparison. Scale bars: 50 μm. Intracellular levels of (b) calcium and (c) ROS were measured by the fluorescence intensity with a microplate reader. Quantitative results were the mean of three independent experiments. (d) Apoptotic index of RCKs exposed to various SRCN samples determined by the TUNEL assay. Values are mean ± SD (n = 6); $^*P < 0.05$ vs all groups; $^{\#}P < 0.05$ vs HP, 2 N, and 4 N groups. (e) TNF-α released from RCK cultures after incubation with various SRCN samples for 24 h. Values are mean ± SD (n = 5); $^*P < 0.05$ vs all groups. The percent of (f) HUVEC proliferation and (g) migrated cells after a 24 h-exposure to VEGF-A_{165} and tested materials. Representative microscopic images of VEGF-A_{165}-induced (h) HUVEC migration and (i) tube formation after a 24 h-exposure to different types of SRCNs; Ctrl: without SRCNs. (j) The percent of tube formation after a 24 h-exposure to VEGF-A_{165} and tested materials. (k) The vascular density index of corresponding CAMs after 24 h of exposure to cellulose filter paper discs pretreated with test samples. The Ctrl group in the CAM assays is cellulose filter paper discs without materials (PBS only). Values are mean ± SD (n = 5); $^*P < 0.05$ vs all groups. The relative expression level of (l) p-IκB-α in the whole cell lysates and (m) NF-κB in the nucleus using ImageJ

software. Values are mean ± SD ($n = 4$); $*P < 0.05$ vs all groups; $^{\#}P < 0.05$ vs IL-1β, 2 N, and 4 N groups.

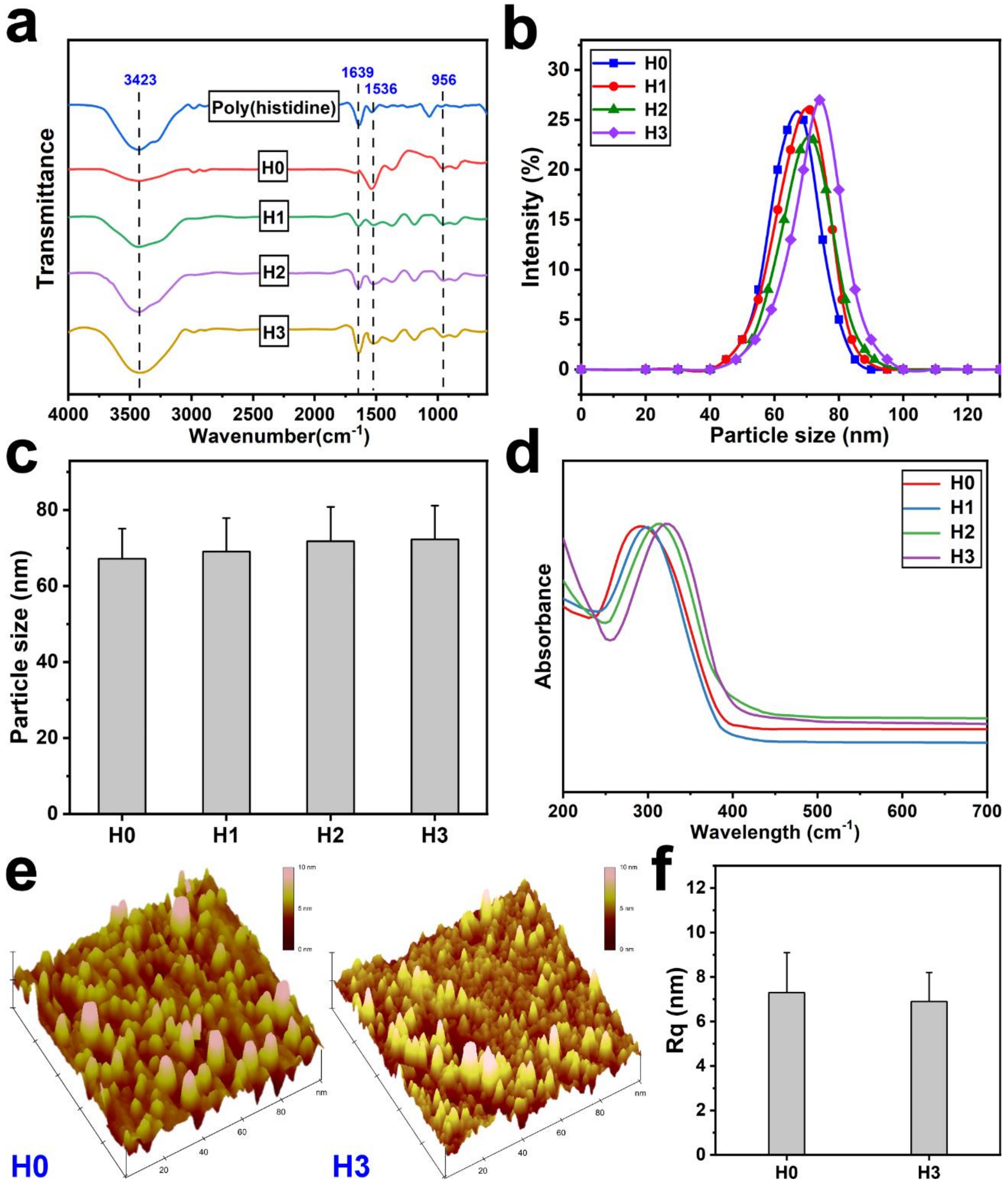

Figure S6. (a) FTIR spectra, (b) DLS profiles, (c) particle diameters (*n* = 5), (d) UV-visible absorption spectra (e) surface texture, and (f) Rq of the SRCNs coated without/with poly(L-histidine). Values are mean ± SD (*n* = 4).

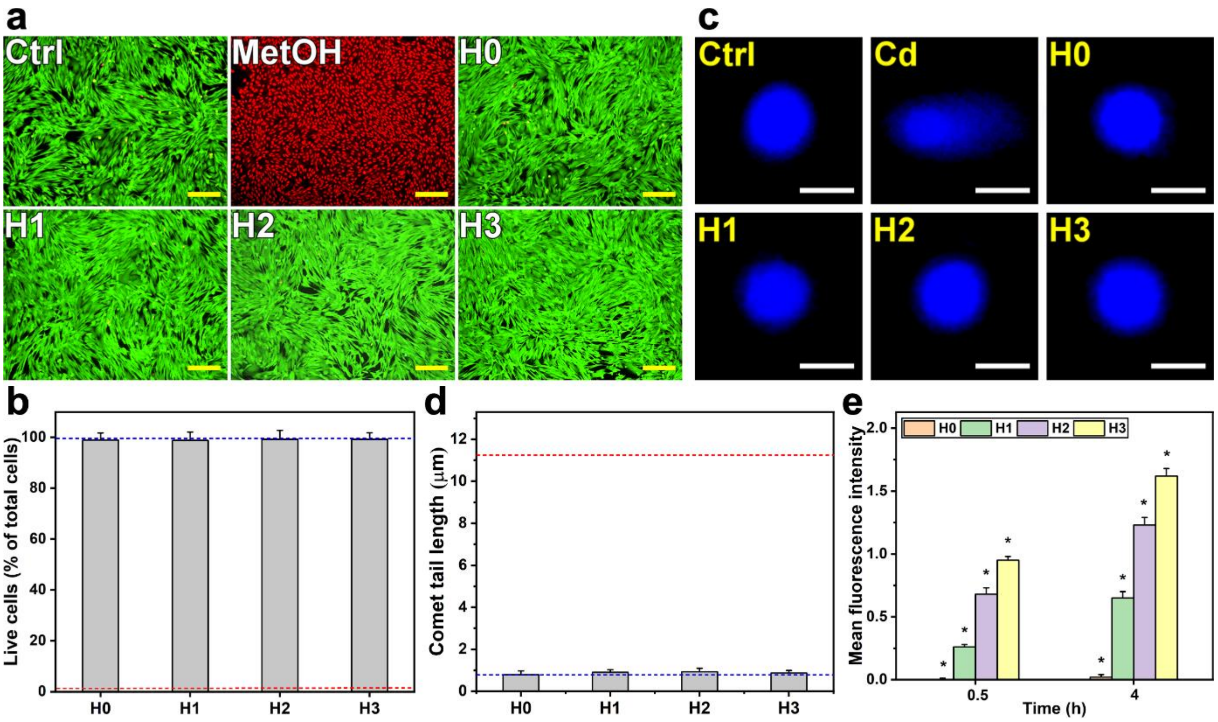

Figure S7. (a) Fluorescence photomicrographs and (b) live cells (live/dead assay) of RCK cultures after a 2-day exposure to the PEGylated/functionalized SRCNs (H0, H1, H2, and H3 samples). The blue and red dash lines represent the values of Ctrl and MetOH groups, respectively. Values are mean ± SD (*n* = 5). (c) Fluorescence photomicrographs of comet assay and (d) comet tail lengths of RCKs exposed to the samples for 24 h; Ctrl: without test materials. The blue and red dash lines represent the values of Ctrl and Cd group, respectively. Values are mean ± SD (*n* = 5). (e) Quantitative analysis of fluorescence intensity of the FITC-labelled samples in the cells. Values are mean ± SD (*n* = 5); **P* < 0.05 vs all groups. Scale bars in (a) and (c) are 50 μm and 10 μm, respectively.

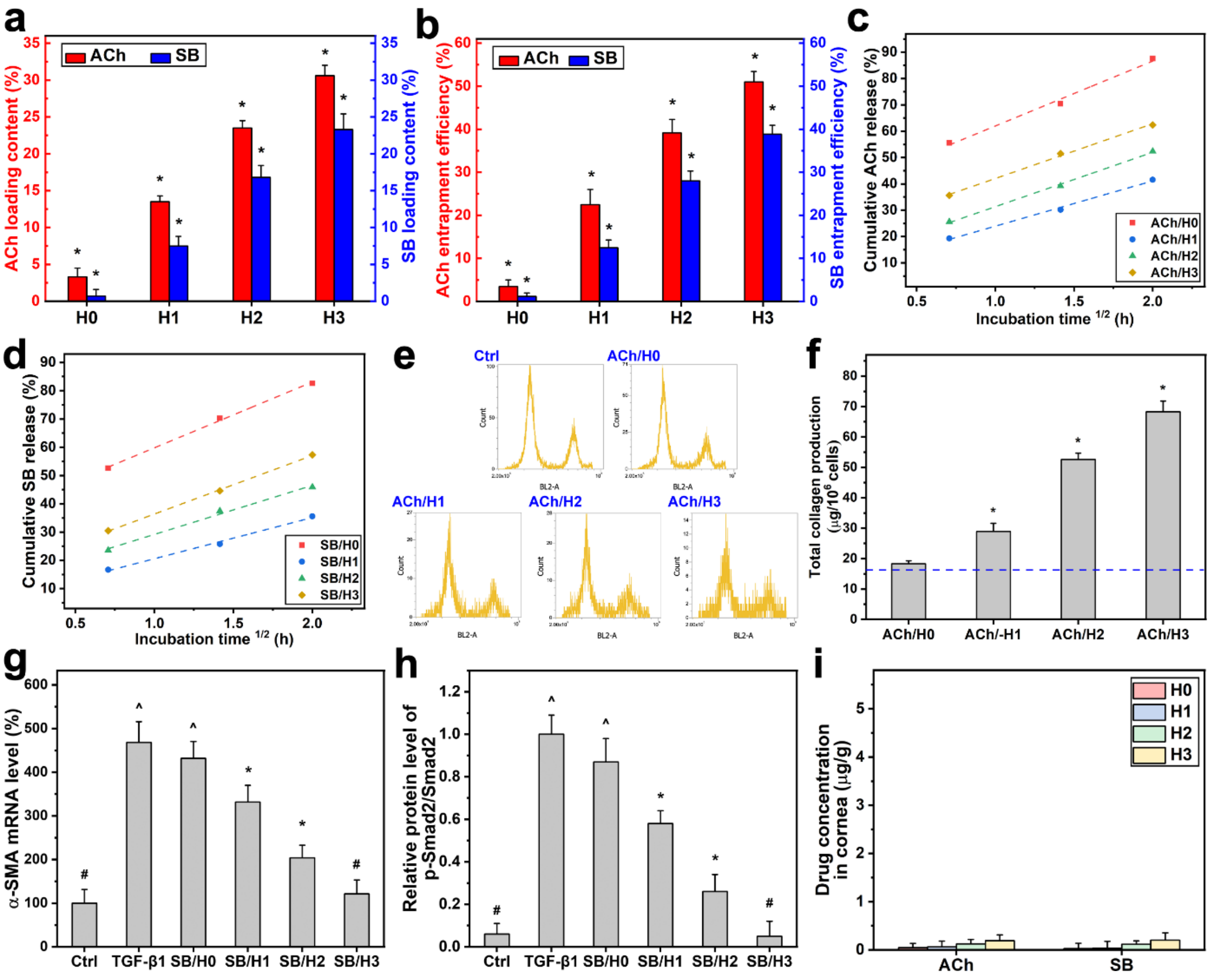

Figure S8. Acetylcholine (ACh) and SB431542 (SB) (a) loading content and (b) entrapment efficiency of the PEGylated/functionalized SRCNs (H0, H1, H2, and H3 samples). Values are mean ± SD (n = 4); *P < 0.05 vs all groups. Cumulative percentage of (c) ACh and (d) SB released from the samples as a function of square root of time. (e) Flow cytometry spectra. (f) Collagen production capacity of RCKs after a 2 day-exposure to the samples containing ACh. The blue dash line represents cells cultured in the absence of test materials (Ctrl group). Results are expressed as percentage of Ctrl. Values are mean ± SD (n = 4); *P < 0.05 vs all groups. (g) α-SMA mRNA level of TGF-β1-induced RCK cultures treated with different samples containing SB. Values are mean ± SD (n = 6); *P < 0.05 vs all groups; $^{\#}P$ < 0.05 vs TGF-β1, SB/H0, SB/H1, and SB/H2 groups; $^{\wedge}P$ < 0.05 vs Ctrl, SB/H1, SB/H2, and SB/H3 groups. (h) Quantitative analysis of p-Smad2 protein. Values are mean ± SD (n = 4); *P < 0.05 vs all groups; $^{\#}P$ < 0.05 vs TGF-β1, SB/H0, SB/H1, and SB/H2 groups; $^{\wedge}P$ < 0.05 vs Ctrl, SB/H1, SB/H2, and SB/H3 groups. (i) Drug (ACh and SB) concentration in the cornea of healthy eyes treated with the samples containing the drugs at 4 days post-administration. Values are mean ± SD (n = 4).

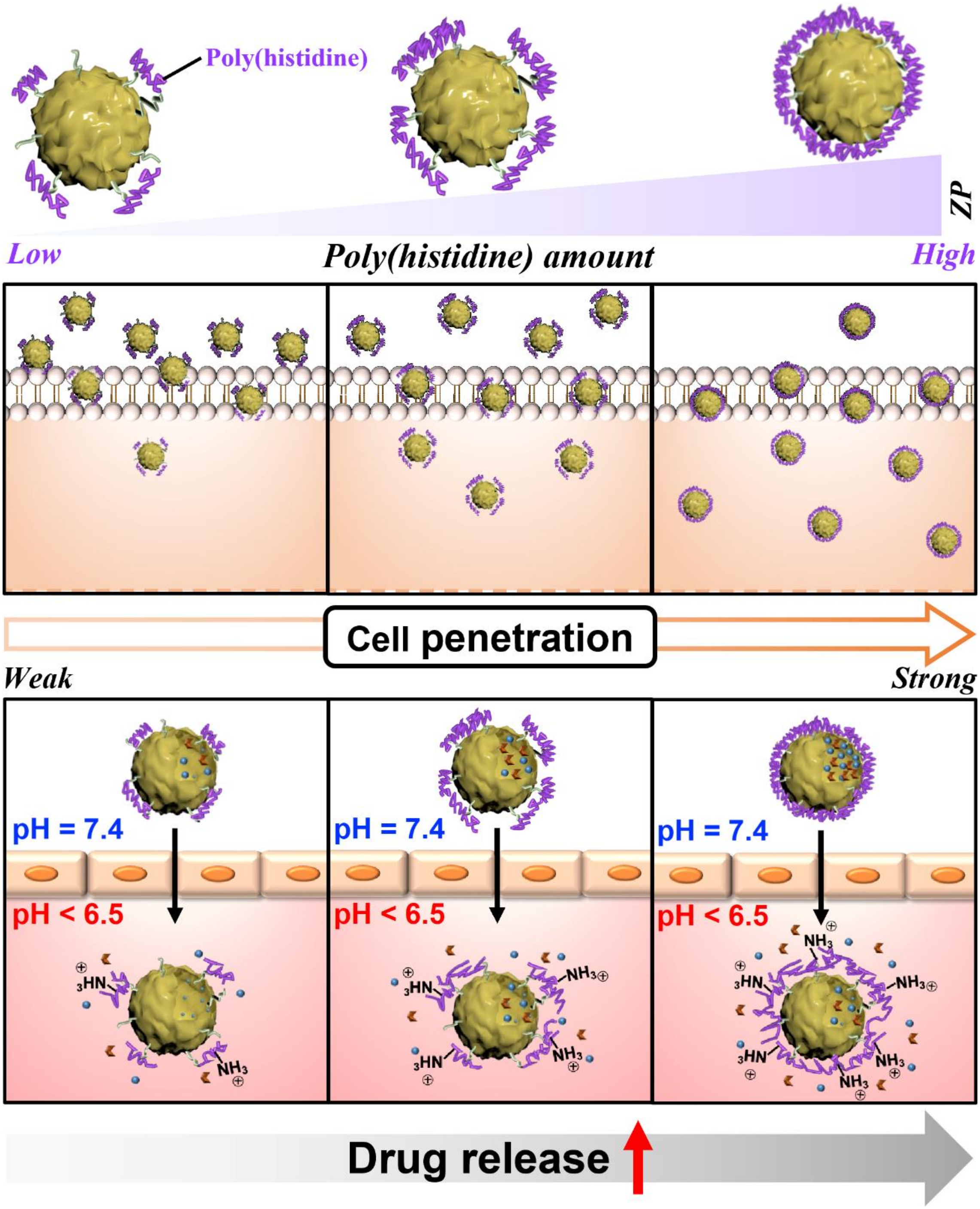

Figure S9. Schematic illustration of the SRCNs coated with different amounts of poly(L-histidine), demonstrating the increase in cell penetration and drug release capacities of the nanocarriers with increasing the polypeptide content.

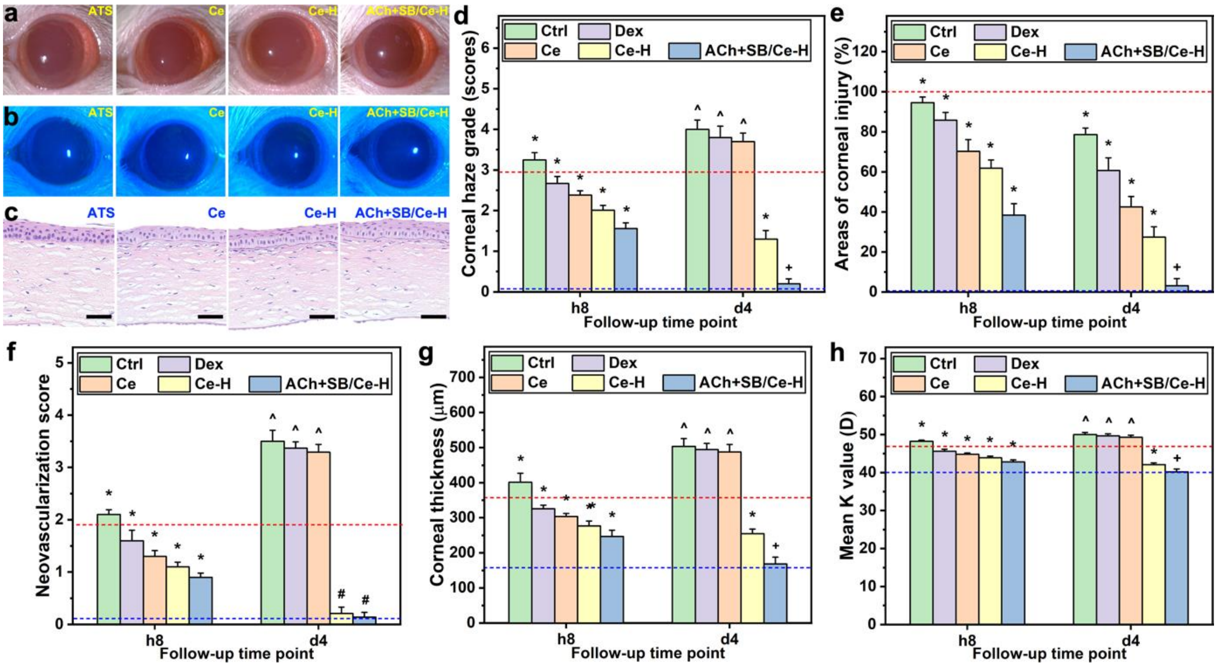

Figure S10. (a) Typical slit-lamp biomicroscopic, (b) corneal fluorescein staining, and (c) corneal H&E staining images of healthy rat eyes at 4 days post-instillation of ATS and ACh+SB/Ce-H nanoformulation and its components including SRCNs (Ce) and poly(L-histidine) functionalized SRCNs (Ce-H), respectively. Scale bars in (c) are 50 μm. (d) Corneal haze grade, (e) corneal wound area, (f) corneal neovascularization score, (g) corneal thickness, and (h) mean K value of alkali burn (AB) eyes at 8 h and 4 d post-instillation of ATS (Ctrl), dexamethasone eye drop (Dex), Ce, Ce-H, and ACh+SB/Ce-H, respectively. The blue and red dash lines represent the value of Pre and AB groups. Values are mean ± SD (n = 10); *P < 0.05 vs all groups; $^{\wedge}P$ < 0.05 vs Pre, AB, Ce-H, and ACh+SB/Ce-H groups; ^{+}P < 0.05 vs AB, Ctrl, Dex, Ce, and Ce-H groups; $^{\#}P$ < 0.05 vs AB, Ctrl, Dex, and Ce groups.

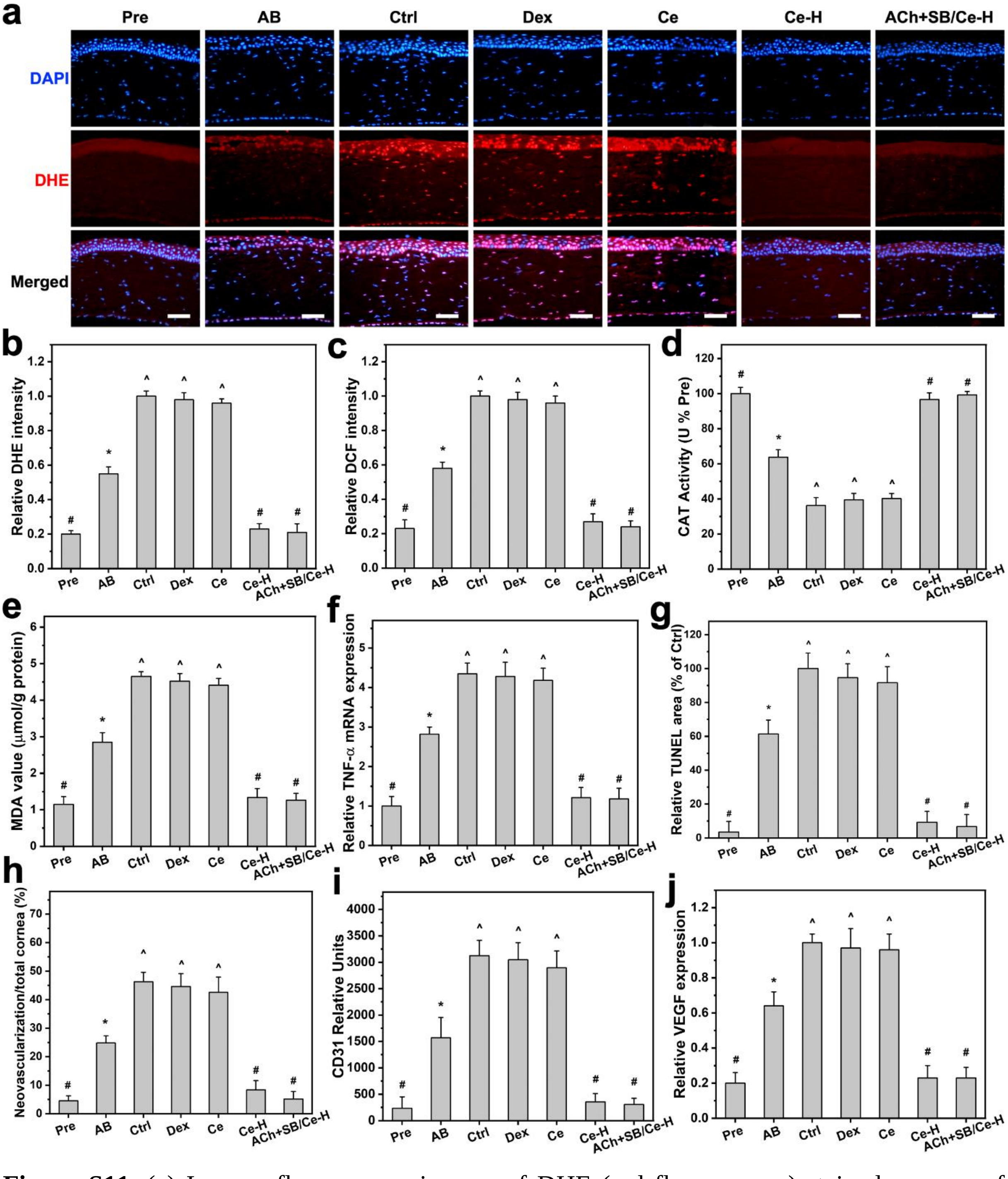

Figure S11. (a) Immunofluorescence images of DHE (red fluorescence)-stained corneas of AB rat eyes instilled with ATS (Ctrl), dexamethasone eye drop (Dex), Ce, Ce-H, and ACh+SB/Ce-H, respectively. Scale bars: 50 μm. Relative (b) DHE and (c) DCF intensity, (d) CAT activity, (e) MDA value, (f) TNF-α levels, (g) TUNEL area, (h) quantification of vascularized area to total corneal areas, and (i) CD31 relative units of corneas in the AB rat eyes at 4 days post-instillation of ATS (Ctrl), dexamethasone eye drop (Dex), Ce, Ce-H, and ACh+SB/Ce-H, respectively. (j) The relative expression level of VEGF using ImageJ software. Values are mean ± SD (n = 10); *P < 0.05 vs all groups; $^{\#}P$ < 0.05 vs AB, Ctrl, Dex, and Ce groups; $^{\wedge}P$ < 0.05 vs Pre, AB, Ce-H, and ACh+SB/Ce-H groups.

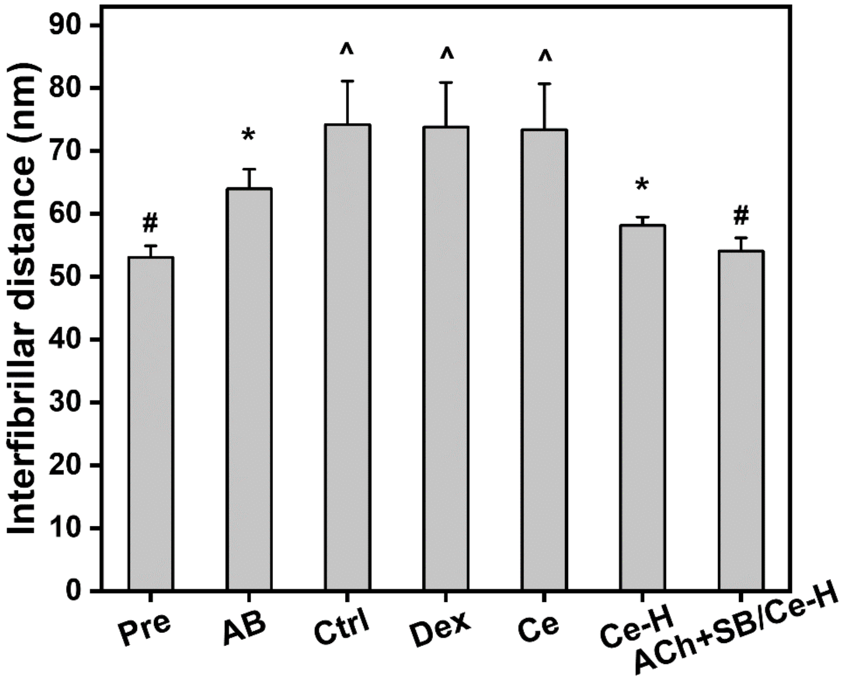

Figure S12. Quantitative results of interspaces between collagen fibrils in the test eyes. Values are mean ± SD (n = 10); *P < 0.05 vs all groups; #P < 0.05 vs AB, Ctrl, Dex, Ce, and Ce-H groups; ^P < 0.05 vs Pre, AB, Ce-H, and ACh+SB/Ce-H groups.

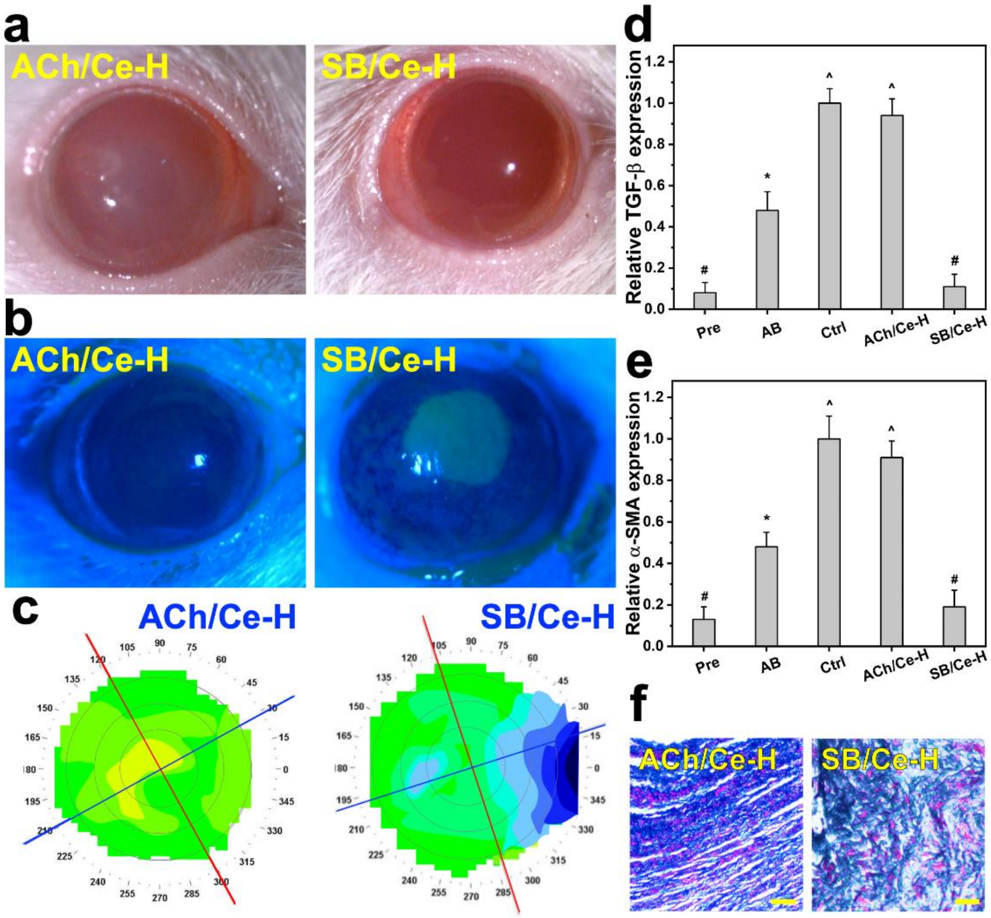

Figure S13. (a) Typical slit-lamp biomicroscopic, (b) corneal fluorescein staining, and (c) corneal topography images of alkali burn eyes at 4 d post-instillation of ACh/Ce-H and SB/Ce-H nanoformulation. (d) TGF-β and (e) α-SMA levels in the corneal tissues of the treated AB eyes. Values are mean ± SD (n = 10); *P < 0.05 vs all groups; $^{\#}P$ < 0.05 vs AB, Ctrl groups, and ACh/Ce-H groups; $^{\wedge}P$ < 0.05 vs Pre, AB, and SB/Ce-H groups. (f) Corneal tissue sections stained with Masson's trichrome. Scale bars are 50 μm.

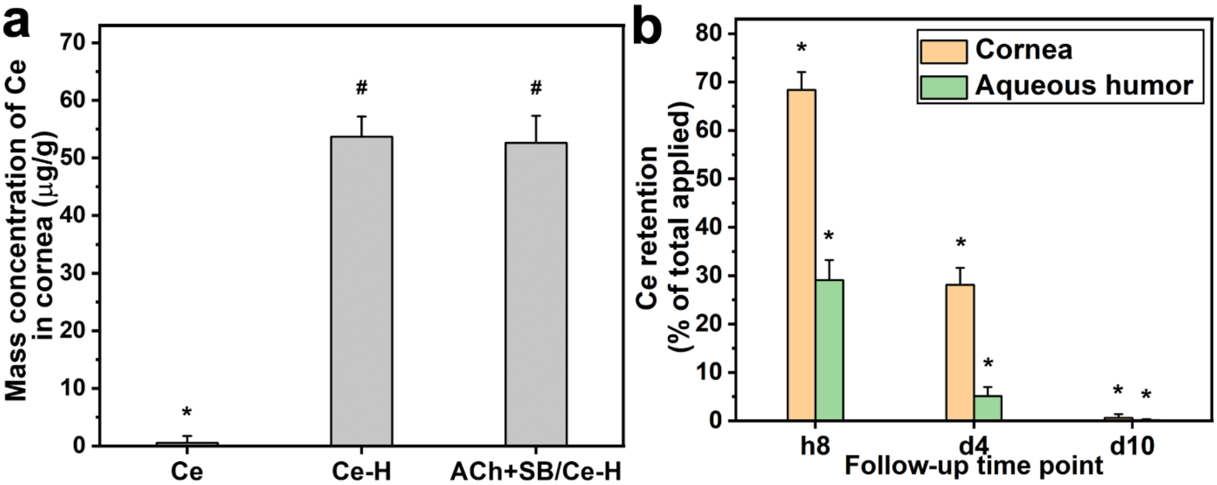

Figure S14. (a) Ce concentration in the cornea of injured eyes treated with the nanoformulations (Ce, Ce-H, and ACh+SB/Ce-H) at 4 days post-administration. Values are mean ± SD (n = 4); *$P < 0.05$ vs all groups; $^{\#}P < 0.05$ vs Ce group. (b) The Ce retention in the corneal tissue and aqueous humor of test rat eyes treated with ACh+SB/Ce-H group at follow-up time points (8 h, 4 d, and 10 d). Values are mean ± SD (n = 10); *$P < 0.05$ vs all groups (compared only within cornea or aqueous humor groups).

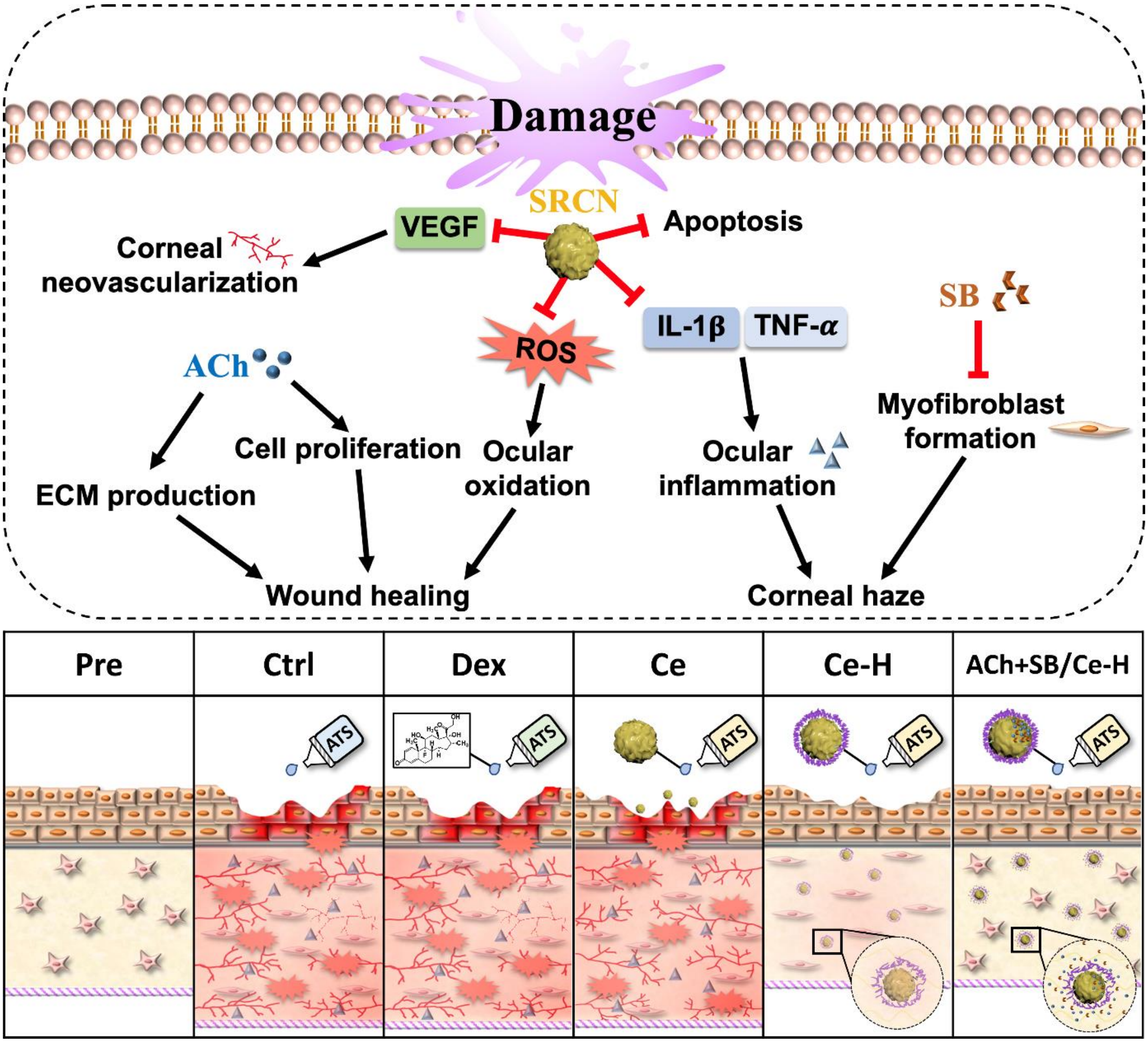

Figure S15. Schematic illustration of the therapeutic and pharmacological activities of the developed ACh+SB/Ce-H nanoformulation with the superior treatment efficacy compared to conventional ophthalmic solutions.
